# Fossil insect‐feeding traces indicate unrecognized evolutionary history and biodiversity on Australia's iconic *Eucalyptus*

**DOI:** 10.1111/nph.20316

**Published:** 2024-11-28

**Authors:** L. Alejandro Giraldo, Peter Wilf, Michael P. Donovan, Robert M. Kooyman, Maria A. Gandolfo

**Affiliations:** ^1^ Department of Geosciences and Earth and Environmental Systems Institute Pennsylvania State University University Park PA 16802 USA; ^2^ Geologic Collections Gantz Family Collections Center, Field Museum of Natural History Chicago IL 60605 USA; ^3^ Department of Biological Sciences Macquarie University Sydney NSW 2109 Australia; ^4^ Research Centre for Ecosystem Resilience Royal Botanic Gardens Sydney NSW 2000 Australia; ^5^ Missouri Botanical Garden St. Louis MO 63110 USA; ^6^ LH Bailey Hortorium, Plant Biology Section, School of Integrative Plant Science Cornell University Ithaca NY 14853 USA; ^7^ Museo Paleontológico Egidio Feruglio Trelew 9100 Chubut Argentina

**Keywords:** eucalypts, Gondwana, hidden biodiversity, host‐tracking, leaf mining, Myrtaceae, niche conservatism, paleobotany

## Abstract

- Fossilized plant–insect herbivore associations provide fundamental information about the assembly of terrestrial communities through geologic time. However, fossil evidence of associations originating in deep time and persisting to the modern day is scarce.
- We studied the insect herbivore damage found on 284 *Eucalyptus frenguelliana* leaves from the early Eocene Laguna del Hunco rainforest locality in Argentinean Patagonia and compared damage patterns with those observed on extant, rainforest‐associated *Eucalyptus* species from Australasia (> 10 000 herbarium sheets reviewed).
- In the fossil material, we identified 28 insect herbivory damage types, including 12 types of external feeding, one of piercing‐and‐sucking, five of galls, and 10 of mines. All 28 damage types were observed in the herbarium specimens.
- The finding of all the fossil damage types on extant *Eucalyptus* specimens suggests long‐standing associations between multiple insect herbivore lineages and their host genus spanning 52 million years across the Southern Hemisphere. This long‐term persistence, probably enabled through niche conservatism in wet eucalypt forests, demonstrates the imprint of fossil history on the composition of extant insect herbivore assemblages. Although the identities of most insect culprits remain unknown, we provide a list of *Eucalyptus* species and specific population locations to facilitate their discovery, highlighting the relevance of fossils in discovering extant biodiversity.

## Introduction

Plants and their insect herbivores represent the bulk of terrestrial, nonmicrobial biodiversity, accounting for more than half of all described species (Futuyma & Agrawal, 2009). This remarkable richness has been generally ascribed to the evolutionary arms race between plants and their associated insect herbivores, wherein producers and consumers reciprocally drive evolutionary change and subsequent bursts of speciation (Ehrlich & Raven, 1964; Futuyma & Agrawal, 2009; Endara *et al*., 2017). As a result, related insects tend to feed on related plants (Ehrlich & Raven, 1964; Futuyma & Agrawal, 2009; Endara *et al*., 2017), and thus, some ancient associations should persist through geologic time. Although fossils show single plant–insect herbivore interactions that have persisted from deep time to the modern day (Opler, 1973; Labandeira *et al*., 1994; Wilf *et al*., 2000; Winkler *et al*., 2010; Leckey & Smith, 2015; Su *et al*., 2015; Adroit *et al*., 2020), evidence for the enduring presence of multiple associations on a single plant host lineage is extremely scarce (Donovan *et al*., 2020, 2023), and the imprint of fossil history on the composition of extant insect herbivore assemblages is largely unknown.

The iconic genus *Eucalyptus* (gum trees, Myrtaceae) provides a rare opportunity to explore how insect herbivores assembled on a plant host through geologic time, due to its high extant diversity (> 700 species; Hill *et al*., 2016), ecological dominance in the Australian landscape (Wrigley & Fagg, 2010), and long fossil history starting in Patagonia during the early Eocene (Gandolfo *et al*., 2011; Hermsen *et al*., 2012; Zamaloa *et al*., 2020). The extant diversity of *Eucalyptus* is heavily concentrated in Australia, although some species are naturally distributed in Papua New Guinea, East Timor, Indonesia, and the Philippine island of Mindanao (Pryor & Johnson, 1981; Williams & Woinarski, 1997; Ladiges *et al*., 2003). Growing from coastal to subalpine habitats, and from wet eucalypt forests directly adjacent to or intermixed with rainforests to semi‐arid areas, *Eucalyptus* dominates nearly all of Australia's 65 million hectares of woodlands and *c*. 86% of its 41 million hectares of forests (Ohmart & Edwards, 1991). The genus serves as a food resource to an estimated 15 000–20 000 species of insect herbivores (New, 1983; Majer *et al*., 1997), several herbivorous and sap‐harvesting marsupials, and myriad nectar‐feeding insects, birds and mammals, including many endangered species (Williams & Woinarski, 1997; Woinarski *et al*., 1997; Moore *et al*., 2004). We refer to the often‐tall, humid eucalypt forests as ‘wet eucalypt’ throughout the text, noting that in the Australian literature, these are also referred to as ‘wet sclerophyll’ or ‘tall eucalypt’ forests (Ash, 1988). However, ‘wet eucalypt’ is a broader term that is applicable outside Australia and for fossil assemblages where tree height is not known, allowing us to refer to the same niche through time (see the Discussion section).

Despite this modern‐day dominance, the fossil record of *Eucalyptus* is scant and sometimes equivocal, particularly for macrofossils (for reviews, see Rozefelds, 1996; Hill *et al*., 1999, 2016; Hermsen *et al*., 2012; Macphail & Thornhill, 2016). However, the oldest, most complete, and abundant fossil *Eucalyptus* material was described from the early Eocene (52 million years ago (Ma)) Laguna del Hunco (LH) caldera‐lake locality in northwestern Chubut, Argentinean Patagonia, which preserves one of the most diverse Eocene floras world‐wide (Wilf *et al*., 2003; Gandolfo *et al*., 2011). The *Eucalyptus* fossils – consisting of leafy branches and isolated leaves with abundant and diverse insect herbivore damage; a flower with *in situ* pollen, flower buds, infructescences, and dispersed capsules – are confidently placed within crown group *Eucalyptus* based on many distinctive features, including leaf architecture, oil glands, operculate flower buds, infructescence structure, and valvate capsulate fruits (Gandolfo *et al*., 2011; Hermsen *et al*., 2012; Zamaloa *et al*., 2020). The presence of a transverse scar on the flower buds most likely indicates that the fossils belong to the large subgenus *Symphyomyrtus* (Gandolfo *et al*., 2011; Hermsen *et al*., 2012; Zamaloa *et al*., 2020). No insect damage has been reported on *Eucalyptus* (or *Eucalyptus*‐related) foliage from the Cenozoic of Australia and New Zealand (McCoy, 1876; Ettingshausen, 1895; Holmes *et al*., 1982; Pole, 1993, 1994, 2019; Pole *et al*., 1993).

The fossil material from LH shows that *Eucalyptus* had a much broader distribution in deep time, among the once‐extensive rainforests that connected southern Gondwana (Hill, 1994; Wilf *et al*., 2013). Although *Eucalyptus* is not considered a strict rainforest element in Australia (Adam, 1994; Tng *et al*., 2012b), in Eocene Patagonia, the genus may have inhabited volcanically cleared or landslide areas within or adjacent to rainforests (Gandolfo *et al*., 2011), similar to some extant non‐Australian *Eucalyptus* species that colonize areas affected by landslides and lava flows (Paijmans, 1973; Adam, 1994), or, in modern Australia, that inhabit narrow fringes of wet eucalypt forest at the fire‐disturbed rainforest margins (Harrington *et al*., 2000; Tng *et al*., 2012a,b; Wilf & Kooyman, 2024). The southern Gondwanan rainforests were nearly exterminated following the final separation of South America, Antarctica, and Australia beginning in the early Eocene, which triggered the loss of suitable habitat (Lagabrielle *et al*., 2009; Kooyman *et al*., 2014; Dunn *et al*., 2015). However, *Eucalyptus*, along with many other taxa found at LH that still associate with eucalypts – including ferns, gymnosperms, and angiosperms (Wilf *et al*., 2013, 2019; Rossetto‐Harris *et al*., 2020; Barreda *et al*., 2020; see Discussion section) – survived in Australia, which moved north and eventually collided with Southeast Asia during the late Oligocene, initiating an ongoing biotic interchange (Wilf *et al*., 2003, 2005a, 2013; Hall *et al*., 2011; Kooyman *et al*., 2014, 2019). Areas of Australia that contain wet eucalypt forests, including the Wet Tropics of Queensland and Gondwana Rainforests of Australia World Heritage sites, are renowned for preserving biotas with Gondwanan ancestry, but plants and vertebrates are the best‐known examples (Wet Tropics Management Authority, 2016). The fossil history of *Eucalyptus* frames several important questions: Did specialized insect herbivores follow their plant hosts over these distances? Do ancient Gondwanan insects still feed on these plants?

The LH flora hosted diverse insects, as indicated by rich feeding damage and oviposition traces on leaves (Wilf *et al*., 2005b; Sarzetti *et al*., 2009; Romero‐Lebrón *et al*., 2019) and insect body fossils (Fidalgo & Smith, 1987; Petrulevičius, 2017 and references therein). Moreover, most insect herbivore damage types (DTs) observed in fossils of the conifer *Agathis* (Araucariaceae) from Patagonia (including LH) are also present in extant *Agathis* species in the West Pacific, suggesting that the associated insect herbivore assemblages probably persisted on the survivor plant host lineage through time and space (Donovan *et al*., 2020, 2023). Here, we compared the insect herbivore damage on fossil *Eucalyptus frenguelliana* leaves from LH with that of extant, rainforest‐associated *Eucalyptus* species in Australasia to evaluate whether insect herbivore assemblages tracked the iconic angiosperm genus for 52 Ma, and to assess whether fossil insect damage can help in discovering extant insect diversity.

## Materials and Methods

### Fossil *Eucalyptus* data collection

**Fig. 1:**
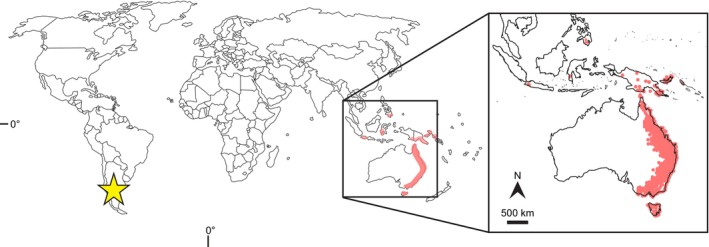
Geographic location of the early Eocene Laguna del Hunco locality (yellow star), the source of the fossil *Eucalyptus frenguelliana* material, alongside the distribution of extant rainforest‐associated *Eucalyptus* species reviewed from herbarium specimens (pink areas) for insect herbivore damage comparable with the fossils. Maps modified from the Australasian Virtual Herbarium (https://avh.chah.org.au/).

This work surveyed 284 *E. frenguelliana* Gandolfo & Zamaloa leaf fossils from the early Eocene Laguna del Hunco (LH) locality in the Huitrera Formation, which crops out near 42.5°S, 70°W in northwest Chubut province, Patagonian Argentina (Fig. 1), collected during multiple international expeditions run from the Museo Paleontológico Egidio Feruglio (MEF; Trelew, Chubut Province, Argentina) from 1999 to 2023 (Wilf *et al*., 2003, 2005a; Gandolfo *et al*., 2011; Hermsen *et al*., 2012). The Tufolitas Laguna del Hunco, a tuffaceous sedimentary unit of the Huitrera Formation exposed at LH, is a highly fossiliferous caldera‐lake deposit (Aragón & Mazzoni, 1997; Wilf *et al*., 2003; Gosses *et al*., 2021) with numerous individual quarries defined in Wilf *et al*. (2003) in a vertical interval of *c*. 60 m in the lower middle part of the 170‐m local section (Wilf *et al*., 2005a). The *Eucalyptus* material comes from 17 individual quarries (Supporting Information Dataset S1). Geochronologic constraints from ^40^Ar/^39^Ar analyses of sanidine crystals from primary airfall tuffs have yielded an age of 52.54 (±0.17) Ma for the uppermost strata of the underlying Barda Colorada ignimbrite, setting a maximum age for all the LH fossils, as well as an age of 52.22 (±0.22) Ma in the middle of the fossiliferous horizons, a date that is routinely used to approximate the age of the entire flora (Wilf *et al*., 2003, 2005a; Wilf, 2012; Gosses *et al*., 2021). All fossil specimens are curated in the Paleobotany Collection of the MEF (repository acronym MPEF‐Pb). Specimens were photographed at MEF using a Nikon D700 and other DSLR cameras, and microphotography was done using a Nikon Eclipse 50i compound microscope with a Nikon DSFi3 camera and DS‐L4 tablet controller. Reversible image adjustments, such as white balance, temperature, and contrast, were performed using Adobe Camera Raw v.15.1.1. An image library of the *E. frenguelliana* collection is available in FigShare (doi: 10.6084/m9.figshare.24756975).

### Damage type scoring

**Fig. 2:**
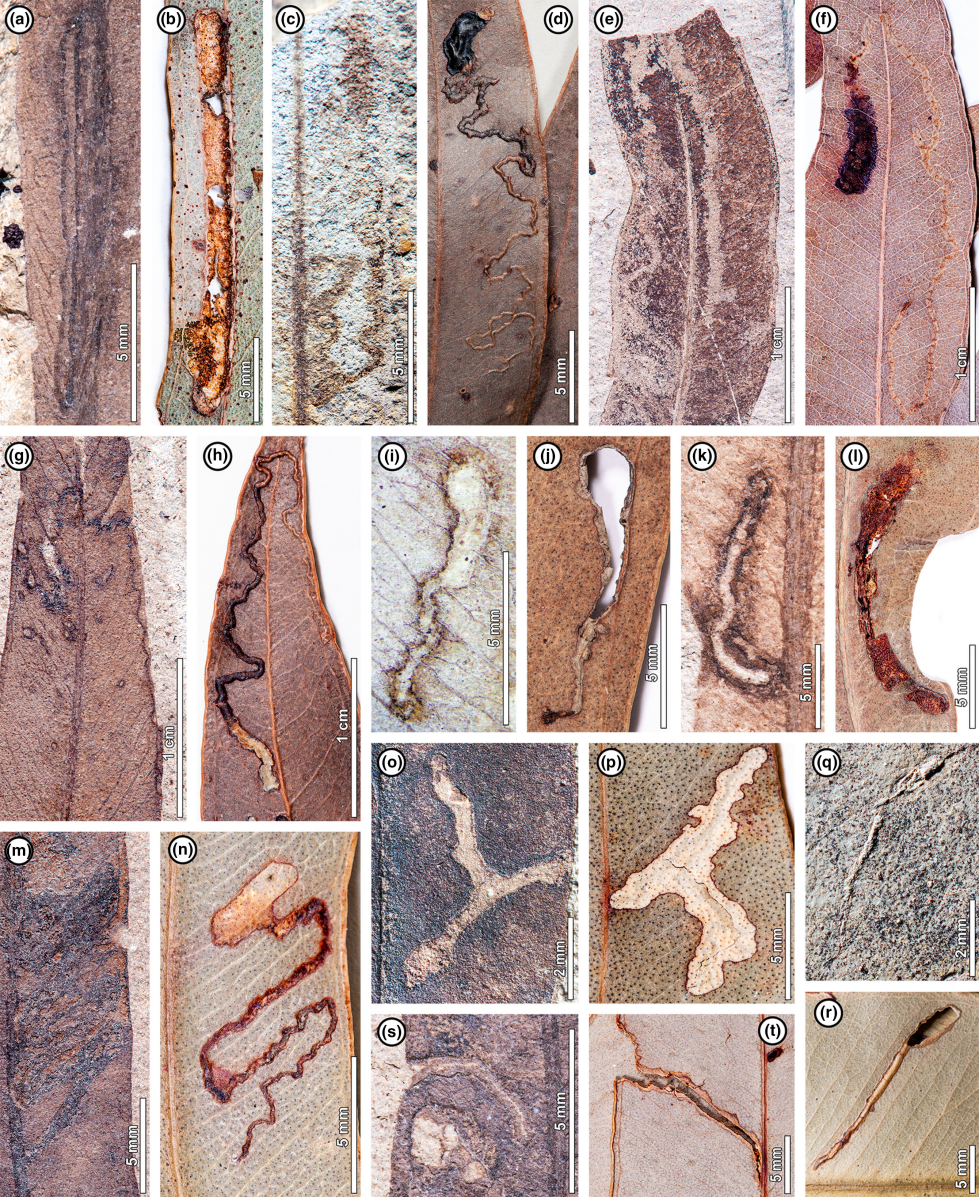
Paired examples of mining in fossil *Eucalyptus frenguelliana* leaves from Laguna del Hunco (first of pair) and corresponding analogs on extant *Eucalyptus* species (second of pair). Note that the paths of the mines begin narrow and progressively widen, thus indicating the directions of the mines. Museum codes for the fossils and catalogue codes for the herbarium specimens are given in Supporting Information Notes S1 and Table S1. (a, b) Mines occurring alongside the midvein, with constant width (newly described DT422) (b *E. tereticornis*). (c, d) Thin, highly serpentine mines with elliptical terminal chambers (DT171) (d *E. cloeziana*). (e, f) Serpentine mines crossing the midvein (DT94) (f *E. decolor*). (g, h) Thread‐like mines with wider phase after crossing the midvein (DT41) (h *E. cloeziana*). (i, j) Short, curvilinear mines deeply embedded into the leaf tissue (DT139) (j *E. melliodora*). (k, l) Short, curvilinear mines with massive reaction tissue and associated leaf shape deformation (DT185) (l *E. crebra*). (m, n) Strongly folded serpentine mines, with paths determined by secondary veins (DT92) (n E. major). (o, p) Bifurcating mines with smooth borders (DT207) (p *E. punctata*). (q, r) Short mines circumscribed by secondary veins and elliptical terminal chambers (DT210) (r *E. robusta*). (s, t) Short, curvilinear mines terminating at the leaf margin (DT90) (t *E. major*).

Each fossil *E. frenguelliana* leaf was inspected for insect‐mediated damage at MEF using a Nikon SMZ1000 binocular microscope and scored for DTs using customized keywords in Adobe Bridge (Rossetto‐Harris *et al*., 2022), following DT definitions as described previously (Labandeira *et al*., 2007 and subsequent addenda). We revised and updated DT designations made by Wilf *et al*. (2005b), resulting in four extra DTs and a newly defined DT422 (Fig. 2a,b), whose detailed description is in preparation elsewhere. The *E. frenguelliana* leaf fossils documented by Wilf *et al*. (2005b), not formally recognized until later (Gandolfo *et al*., 2011), were treated at the time as morphotype TY21, ‘*Myrcia chubutensis*’ (Hermsen *et al*., 2012). We also scored all subsequent *E. frenguelliana* fossils collected during later field seasons up to 2023. The DTs were assigned to corresponding functional feeding groups, including external feeding (encompassing hole, margin, and surface feeding, as well as skeletonization), piercing‐and‐sucking, galling, and mining (Labandeira *et al*., 2007). The *E. frenguelliana* fossils also have oviposition marks (Sarzetti *et al*., 2009; Romero‐Lebrón *et al*., 2019) and pathogenic traces, although our focus here is on insect herbivory. Measurements were performed using ImageJ (Schneider *et al*., 2012). A complete catalog of specimens and their corresponding quarries, DTs, and field and museum numbers, is available in Dataset S2.

### Extant *Eucalyptus* data collection

We selected 36 extant, rainforest‐associated *Eucalyptus* species and examined more than 10 000 herbarium specimens of those species for insect herbivore damage comparable with the fossils (Dataset S3). Most of the reviewed herbarium sheets are from eastern Australia, with fewer specimens originating from Papua New Guinea, Indonesia, and the island of Mindanao in the Philippines (Fig. 1). We followed the most recent phylogeny of *Eucalyptus* (Thornhill *et al*., 2019), based on the taxonomy of Nicolle (2015), and defined rainforest‐associated *Eucalyptus* as those species whose ranges include wet habitats, frequently in narrow fringes of wet eucalypt vegetation beside rainforests (see the Discussion section; Harrington & Sanderson, 1994; Tng *et al*., 2012a,b). However, the reviewed herbarium specimens span the full range of habitats for a given species. We chose rainforest‐associated taxa (Dataset S3) because their ecology includes close matches to LH, wherein *Eucalyptus* probably colonized disturbed areas within or adjacent to intact rainforests (Gandolfo *et al*., 2011; see the Discussion section).

We studied all the available material (*c*. 7400 herbarium sheets) from these 36 species in‐person at the Australian National Herbarium, Canberra (CANB and CBG), the Queensland Herbarium and Biodiversity Science, Brisbane (BRI), and the Harvard University Herbaria, Cambridge (A and GH). Photographs were taken using a Nikon D700 DSLR with a 105 mm f/2.8D lens and polarizing filter mounted on a minitripod, together with two Nikon SB‐R200 wireless miniflashes. We also examined *c*. 3100 digitized, high‐resolution herbarium sheets of the 36 selected species from several Australian herbaria, including the National Herbarium of New South Wales of the Royal Botanic Gardens and Domain Trust, Mount Annan (NSW), and the Royal Botanic Gardens Victoria, Melbourne (MEL) (both through the Australasian Virtual Herbarium; https://avh.chah.org.au/); along with the Naturalis Biodiversity Center, Leiden (L, U, WAG, and AMD; https://bioportal.naturalis.nl/), the Royal Botanic Garden Edinburgh (E; https://data.rbge.org.uk/search/herbarium/), Royal Botanic Gardens Kew, Richmond (K; http://apps.kew.org/herbcat/navigator.do), the Muséum National d'Histoire Naturelle, Paris (P; https://science.mnhn.fr/institution/mnhn/item/search/form), the United States National Herbarium of the Smithsonian Institution, Washington D.C. (US; https://collections.nmnh.si.edu/search/botany/), and The New York Botanical Garden, New York (NY; https://sweetgum.nybg.org/science/vh/).

### Extant *Eucalyptus* insect herbivores

We surveyed relevant entomological, ecological, and forestry literature to gather unique records of insect herbivore species associated with *Eucalyptus* hosts. We report *c*. 1700 plant–insect feeding associations in Dataset S4, along with updated taxonomy for both insects and *Eucalyptus* species, a classification of the associations following the functional feeding group approach outlined previously (Labandeira *et al*., 2007), and whether or not the association was illustrated (drawing or photograph). For scale insect–host association data, we particularly used ScaleNet (García Morales *et al*., 2016). We consulted all the cited literature in order to verify host association range and view original descriptions and illustrations. We limited our search to regions where *Eucalyptus* species are naturally distributed, but we rarely included records outside these areas if the insect herbivore was introduced from its native range (e.g. several gall‐inducing hymenopterans; Dittrich‐Schröder *et al*., 2020). This survey represents, to the best of our knowledge, the most comprehensive dataset of associations between *Eucalyptus* species and their insect herbivores.

## Results

We identified 28 DTs on 284 fossil *E. frenguelliana* leaves, including 10 types of mines, five of galls, one of piercing‐and‐sucking marks, and 12 of external feeding associations (including hole, margin, and surface feeding and skeletonization; see the Materials and Methods section). Nine of the 28 DTs are exclusive to *E. frenguelliana* within the highly diverse LH flora's damage assemblage (Wilf *et al*., 2005b), including seven mine, one gall, and one piercing‐and‐sucking DTs (Table S1), indicating host specialization.

**Fig. 3:**
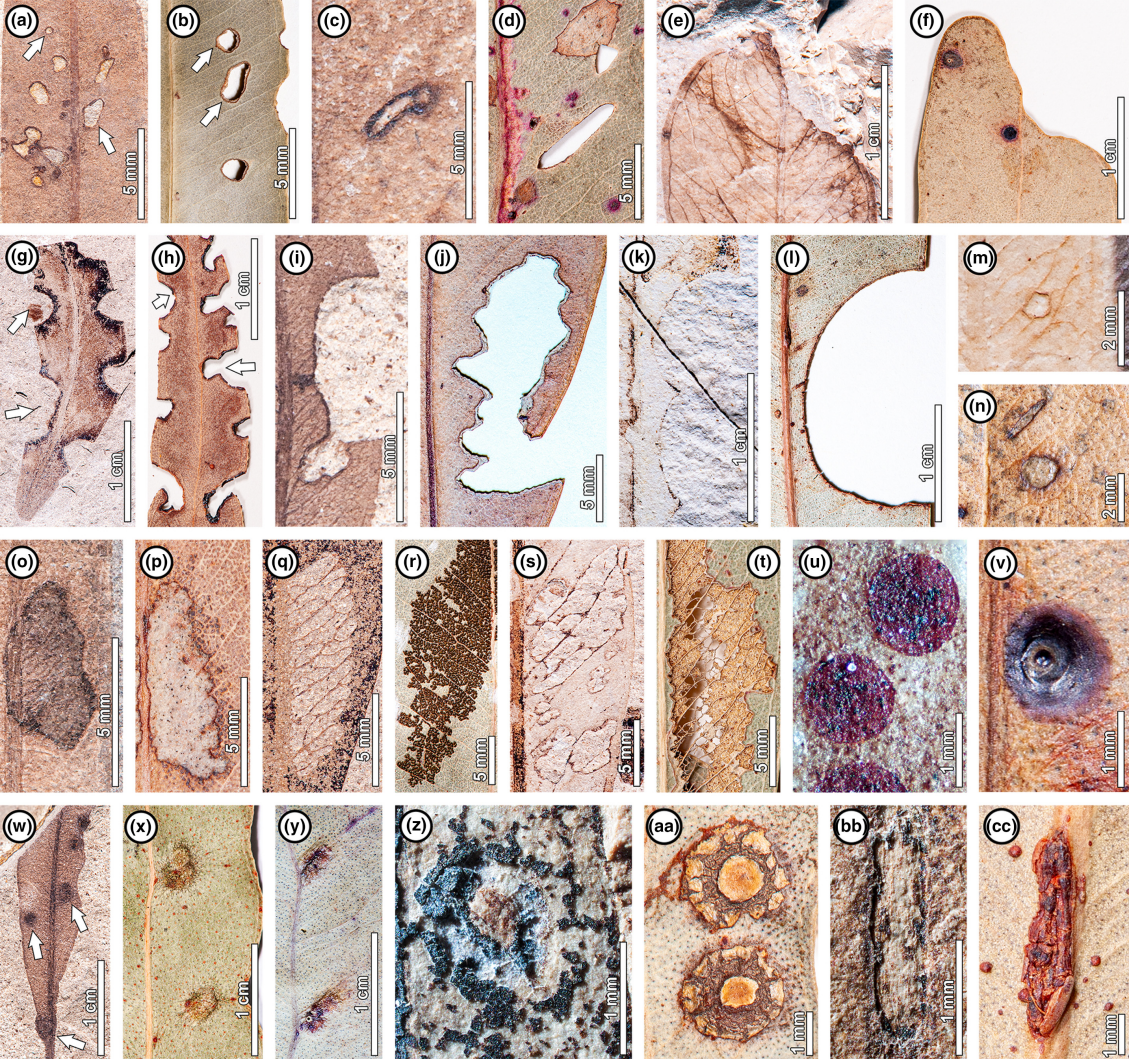
Paired examples of hole feeding (a–d), margin feeding (e–l), surface feeding (m–p), skeletonization (q–t), piercing‐and‐sucking (u–v), and galling (w–cc) in fossil *Eucalyptus frenguelliana* leaves from Laguna del Hunco (first of pair) and corresponding analogs on extant *Eucalyptus* species (second of pair). Museum codes for the fossils and catalogue codes for the herbarium specimens are given in Supporting Information Notes S1 and Table S1. (a, b) Small circular (DT1; uppermost arrows) and polylobate (DT3; lowermost arrows) holes (b *E. resinifera*). (c–d) Elongate holes (DT8) (d *E. major*). (e, f) Excisions removing leaf apices (DT13) (f *E. crebra*). (g, h) Excisions into the leaf margin, either shallow (DT12; uppermost arrows) or reaching the midvein (DT14; lowermost arrows) (h *E. acmenoides*). (i, j) Deeply trenched excisions (DT15) (j *E. fibrosa*). (k, l) Consecutive, nearly perfect semicircular excisions along the leaf margin (DT81) (l *E. grandis*). (m, n) Circular surface abrasions with thick reaction rims (DT31) (n *E. robusta*). (o, p) Polylobate surface abrasions with thick reaction rims (DT30) (p *E. fibrosa*). (q, r) Skeletonized areas without reaction rims (DT16) (r *E. michaeliana*). (s, t) Skeletonized areas with thick reaction rims (DT17) (t *E. tereticornis*). (u, v) Circular scale insect (Diaspididae) covers occurring in clusters (DT77) (v *E. notabilis*). (w–y) Featureless galls occurring in the leaf lamina (DT32; middle arrow in w and lowermost gall in x), alongside the midvein (DT33; lowermost arrow in w and galls in y), or alongside secondary veins (DT34; uppermost arrow in w and uppermost gall in x) (x *E. punctata*; y *E. propinqua*). (z, aa) Thick, ellipsoidal galls with internal carbonized cores (DT49) (aa *E. punctata*). (bb, cc) Lenticular galls occurring along midvein (DT85) (cc *E. tereticornis*).

**Fig. 4:**
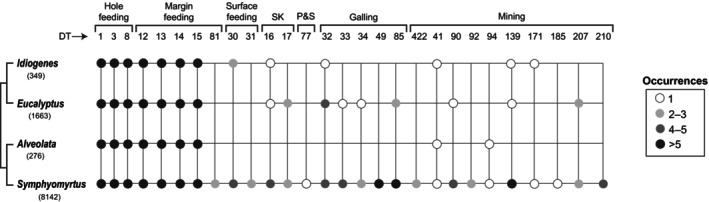
Occurrences of insect herbivory damage types found in *Eucalyptus frenguelliana* leaf fossils from Laguna del Hunco (columns) on extant *Eucalyptus* subgenera, from our herbarium survey. Phylogenetic relationships among subgenera are summarized from Thornhill *et al*. (2019; four unsampled subgenera not included). Labels beneath subgenera indicate the number of herbarium sheets reviewed for that subgenus (Supporting Information Dataset S3). SK, skeletonization; P&S, piercing‐and‐sucking.

From our survey of > 10 000 herbarium specimens pertaining to 36 extant, rainforest‐associated *Eucalyptus* species, we found a corresponding analog for each fossil DT (Figs 2, 3; Notes S1; Table S1). Shared morphological features between fossil insect damage and corresponding extant analogs include size, shape, position, callus tissue development, as well as (in the case of mines) direction, oviposition site, terminal chamber, and frass accumulation pattern. We observed that the complete suite of 28 DTs recorded from the fossils is only seen among living species of *Eucalyptus* subgenus *Symphyomyrtus* (Fig. 4; Table S1), consistent with the proposed affinities of the fossil plant material (Gandolfo *et al*., 2011; Hermsen *et al*., 2012; Zamaloa *et al*., 2020). However, this result could be an artifact of sample size (Fig. S1), given that 26 of the 36 extant species surveyed and *c*. 72% of the reviewed herbarium sheets pertain to *Symphyomyrtus* (Dataset S3).

**Fig. 5:**
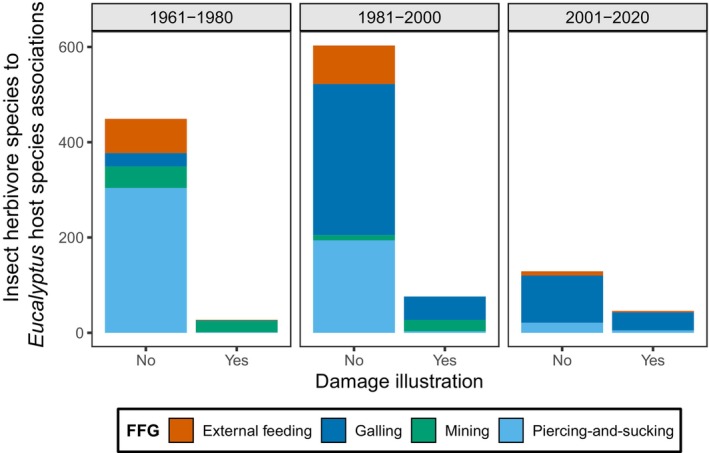
Literature survey totals of representative species‐level associations of insect herbivores and *Eucalyptus* host plants described vs described and illustrated (drawing or photograph) from 1961 to 2020. Each bar represents the number of unique insect‐*Eucalyptus* species‐pair associations in the survey (Supporting Information Dataset S4), colored by Functional Feeding Group (FFG; external feeding encompasses hole, margin, and surface feeding, as well as skeletonization) following Labandeira *et al*. (2007). Only leaf‐feeding associations are depicted here (i.e. wood boring is excluded), but see Dataset S4 for the full list of associations.

Our survey of the literature revealed that although hundreds of extant insect herbivore species feed on *Eucalyptus*, illustrating feeding traces (either through drawings or photographs) has always been a rare practice (Fig. 5; Notes S2; Dataset S4), greatly limiting our ability to associate insect feeding traces with their insect culprits using the literature alone. Most work on *Eucalyptus* insect herbivores has focused on gall‐inducing and piercing‐and‐sucking eriococcids and psyllids, and, to a lesser extent, externally feeding chrysomelids and lepidopterans (e.g. Moore, 1970; Gullan, 1984; Common, 1990; Tribe & Cillie, 1997; García Morales *et al*., 2016). By contrast, host‐specialized interactions, such as those of mining and gall‐inducing insects, have received far less attention (Hoare *et al*., 1997; Hoare & van Nieukerken, 2013; Dittrich‐Schröder *et al*., 2020) unless they have attained pest status on economically important *Eucalyptus* species. For instance, *c*. 15% of the estimated 150 species of Australian Nepticulidae (Lepidoptera) have been described, and many of the known species mine *Eucalyptus* leaves (Common, 1990; Hoare & van Nieukerken, 2013). Similar numbers of undescribed species are seen across other leaf‐mining lepidopteran families that commonly attack *Eucalyptus* foliage, including Gracillariidae, Heliozelidae, and Incurvariidae (Common, 1990). Conversely, despite being native to Australia, some gall‐inducing hymenopterans were recently described from *Eucalyptus* plantations in Europe, the Middle East, and South America (Mendel *et al*., 2004; Protasov *et al*., 2007; Molina‐Mercader *et al*., 2019; Dittrich‐Schröder *et al*., 2020), and an estimated 320 species of gall‐inducing wasps that attack *Eucalyptus* in Australia still await description (Dittrich‐Schröder *et al*., 2020).

## Discussion

The remarkable preservation of 28 DTs from six functional feeding groups on a single fossil species of *Eucalyptus* suggests that a diverse array of herbivorous insects fed on this plant host, based on the positive among‐species correlation in two extant rainforests between DTs and culprit species richness (Carvalho *et al*., 2014). Although the total number of DTs recorded in *E. frenguelliana* falls in the lower half of the various plant hosts at LH (Wilf *et al*., 2005b), nine of those DTs are exclusive to *E. frenguelliana* at the site (including seven mines, one gall, and one piercing‐and‐sucking mark; Table S1), indicating host specialization. The 10 mining DTs observed in the fossil material appear to represent the highest richness of mines documented for a single plant host in the fossil record, exceeding the number of mine DTs found in many complete fossil floras (Currano *et al*., 2021) and echoing earlier findings of extremely diverse arrays of insect herbivory on single species and even single leaves at LH (Wilf *et al*., 2005b). The only other study that compared suites of insect herbivore damage between fossil and extant specimens within the same host genus found that fossil *Agathis* from four Patagonian sites (including LH) preserved 16 DTs, including three mining DTs (Donovan *et al*., 2020, 2023). The number of DTs recorded in the fossil *E. frenguelliana* leaves is nearly double those observed on fossil *Agathis* (28 vs 16 DTs), and more than triple the number of mining DTs (10 vs 3 DTs), indicating that *E. frenguelliana* was probably utilized by a richer array of herbivorous insects, in accord with general expectations for angiosperm vs conifer herbivore diversity (Labandeira & Wappler, 2023). Nevertheless, these numbers are striking when considering that the *Agathis* material is derived from four localities spanning *c*. 18 Myr (latest Cretaceous to middle Eocene; Donovan *et al*., 2020, 2023) and several hundred kilometers of map distance, whereas all the *E. frenguelliana* leaves are restricted to LH, where the fossil material was probably deposited within *c*. 200 ky (Wilf *et al*., 2023).

The remarkable similarity in insect herbivore damage between fossil and extant *Eucalyptus* (Figs 2, 3) might be attributed to two nonexclusive evolutionary scenarios: host‐tracking and convergence in damage type morphology (Donovan *et al*., 2020). Host‐tracking is more plausible for leaf‐mining and gall‐inducing insects, due to their conservative, host‐specific nature wherein a single insect species creates a morphologically distinct mine or gall on a specific host at a site (Crespi *et al*., 1997; Gonçalves‐Alvim & Fernandes, 2001; Cuevas‐Reyes *et al*., 2004; Labandeira *et al*., 2007; Bairstow *et al*., 2010). Thus, finding the same 10 mining and five galling DTs in both fossil and extant *Eucalyptus* probably indicates that most of the insect culprit lineages have persisted on the host genus through geologic time. In the case of external feeding, which is typically less host‐specific (Carvalho *et al*., 2014), host‐tracking is less certain (though still quite possible) and convergence more likely. The morphological consistency between the scale insect (Diaspididae) covers observed for fossil and extant specimens (Fig. 3u,v) shows that diaspidids have fed on *Eucalyptus* for at least 52 Ma, but it does not necessarily support host‐tracking because multiple diaspidid genera produce similarly shaped covers (Gullan, 1984), and hundreds of scale insect species feed on *Eucalyptus* today (Notes S2; Dataset S4).

Although it is probable that both the host‐tracking and DT‐convergence scenarios operated simultaneously through evolutionary time, the fact that we identified all 28 DTs observed in the fossils in extant *Eucalyptus* species makes the host‐tracking scenario more plausible overall, particularly for the highly diverse mining and galling interactions. A similar rationale was suggested for *Agathis* by Donovan *et al*. (2020, 2023), who found that most of the insect herbivore damage traces observed in fossil specimens were present in extant species of the plant host genus. Interestingly, the full suite of 28 DTs observed in the fossils is only found among extant *Eucalyptus* species in subgenus *Symphyomyrtus*, which is allied with the fossils (Fig. 4; Table S1; Gandolfo *et al*., 2011; Hermsen *et al*., 2012; Zamaloa *et al*., 2020). This signal possibly relates to the distinctive foliar chemistry of symphyomyrts, including their unique formylated phloroglucinol compounds that act as herbivory deterrents and, compared with other subgenera, a higher concentration of available nitrogen and lower tannin content, all of which have been shown to determine the composition of insect and marsupial folivores in extant *Eucalyptus* (Steinbauer & Matsuki, 2004; Moore *et al*., 2004; Wallis *et al*., 2010; Matsuki *et al*., 2011; Jensen *et al*., 2015; Dos Santos *et al*., 2019). However, the existence of this phytochemistry during the Eocene is completely unknown, and the pattern might simply result from our more intensive sampling of *Symphyomyrtus* compared with other *Eucalyptus* subgenera (Fig. S1).

Single plant–insect herbivore interactions have been observed to persist through geologic time (Opler, 1973; Labandeira *et al*., 1994; Wilf *et al*., 2000; Winkler *et al*., 2010; Leckey & Smith, 2015; Su *et al*., 2015; Adroit *et al*., 2020), yet fossil evidence for the continued presence of multiple associations on a single plant host genus from deep time to the present, as described here for *Eucalyptus* and before for *Agathis* (Donovan *et al*., 2020, 2023), is extremely rare. This remarkable host fidelity could result from prolonged environmental stability that allowed plant hosts and their associated insect herbivores to establish long‐term interactions. Plant lineages from LH commonly tracked their preferred rainforest environment from Eocene Patagonia to modern‐day Australasia (Kooyman *et al*., 2014; Merkhofer *et al*., 2015), in accordance with patterns of niche conservatism that have been observed at local, regional, and continental scales (Losos *et al*., 2003; Silvertown *et al*., 2005; Crisp *et al*., 2009). Although extant *Eucalyptus* species are not typically classified as rainforest elements in Australia, several species are closely associated with or found within rainforests (Adam, 1994; Tng *et al*., 2012b). For instance, in temperate Tasmania, *Nothofagus* rainforests often intergrade with *Eucalyptus regnans* and *Eucalyptus obliqua* in what has been termed a mixed forest (Gilbert, 1959). On the other hand, in tropical regions of far north Queensland and subtropical areas in the central coast of Queensland and New South Wales, several *Eucalyptus* species, such as *E. grandis* and *E. pilularis*, commonly inhabit narrow bands of wet eucalypt forests that interact opportunistically with adjacent rainforests (Harrington *et al*., 2000; Tng *et al*., 2012a). In turn, the extra‐Australian *Eucalyptus deglupta* has always been considered a rainforest tree (Tng *et al*., 2012b), frequently occurring on recent lava flows and older volcanic soils of New Guinea (Paijmans, 1973; Johns, 1990). *Eucalyptus frenguelliana* from LH probably had a similar ecology to that of *E. deglupta*, inhabiting volcanically cleared or landslide‐disturbed areas (Gandolfo *et al*., 2011). This rainforest‐associated ecology is further supported by the presence of several plant lineages at LH that today inhabit Australian or New Guinean rainforests and thus frequently interact with *Eucalyptus* species at the disturbance edges, including the fern *Todea*, conifers such as *Agathis*, *Araucaria* (section *Eutacta*), and *Podocarpus*, and angiosperms including *Akania*, *Ceratopetalum*, *Gymnostoma*, *Macaranga*, *Orites*, *Ripogonum,* and close relatives of *Ackama*, *Daphnandra*, *Wilkiea*, and *Zygogynum* (e.g. Wilf *et al*., 2013, 2023; Barreda *et al*., 2020; Matel *et al*., 2022).

Thus, it is probable that the enduring presence of rainforests in the Southern Hemisphere and the likely tracking of the rainforest margin environment by *Eucalyptus* provided sufficient niche stability for the associated insect herbivores to establish long‐term, conservative interactions with their plant host. Subsequent colonization of multiple *Eucalyptus* species (Fig. 4) was probably facilitated by the ecological propinquity of the plant hosts (Futuyma & Agrawal, 2009), or by their chemical similarity (Ehrlich & Raven, 1964; Futuyma & Mitter, 1996; Futuyma & Agrawal, 2009), although the latter idea cannot yet be tested using fossils. Collectively, the pattern of host‐tracking seen here for *Eucalyptus*, and before for *Agathis* (Donovan *et al*., 2020, 2023), reveals the imprint of fossil history on the composition of extant insect herbivore assemblages (Futuyma & Mitter, 1996; Futuyma & Agrawal, 2009). As previously suggested by Winkler *et al*. (2010), relictual associations may be the rule rather than the exception when both the host and insect herbivore lineages have persisted through geologic time.

Although hundreds of extant insect herbivore species are associated with *Eucalyptus* hosts, the number of studies of herbivory has declined rapidly, and very few of those interactions have been illustrated (Fig. 5; Notes S2; Dataset S4), which significantly limits our ability to associate the feeding traces observed in the fossils (and corresponding extant analogs) with their insect culprits using the literature alone (e.g. as in Labandeira *et al*., 1994; Wilf *et al*., 2000; Leckey & Smith, 2015). *Eucalyptus* insect herbivores have been systematically studied since the end of the 19^th^ century (French, 1891), yet research has often focused on pests of commercial timber species (e.g. the jarrah leafminer, *Perthida glyphopa*, on *E. marginata*; Wallace, 1970; Mazanec, 1987; Sinclair & Hughes, 2010). Naturally evolving associations in noncommercial *Eucalyptus* species remain understudied (Sinclair & Hughes, 2008), particularly when the insect herbivore has concealed feeding strategies, such as mining insects (Moore, 1963, 1966; Hoare & van Nieukerken, 2013). However, there is a high potential to discover new insect species with long evolutionary associations with *Eucalyptus* by seeking the extant culprits of the DTs that match those seen in the fossils. To facilitate this opportunity, in Notes S1, we provide the list of herbarium vouchers, whose geospatial data are easily obtained, in which we found DTs that match those seen in the fossils.

In summary, the insect feeding traces observed in the *E. frenguelliana* fossils from Argentine Patagonia indicate unrecognized evolutionary history and biodiversity of herbivorous insects on Australia's iconic *Eucalyptus*. Our finding of the full assemblage of 28 DTs from fossil *Eucalyptus* on extant species suggests that the insect herbivore lineages tracked and radiated on multiple species of their host genus through time (Eocene to the present) and space (Patagonia to Australia and beyond). This persistence was probably facilitated by the sustained presence of rainforests in parts of the Southern Hemisphere and the likely tracking of the dynamic rainforest margin by *Eucalyptus*, which in turn enabled sufficient niche stability for the associated insect herbivores to establish cohesive interactions over long geologic intervals. Although we lack the taxonomic and behavioral information to link most of these feeding traces with their insect culprits using the literature, there is immense potential for discovering new insect species with rich biogeographic histories by searching for the extant culprits of the DTs at the collection locations of the herbarium vouchers. This work highlights the importance of deep time–modern comparisons and the relevance of natural history collections and observations, from fossil and herbarium specimens to documenting and illustrating insect herbivore–plant host associations, in testing evolutionary questions.

## Competing interests

None declared.

## Author contributions

LAG, PW and RMK designed research. LAG, PW, MPD and MAG conducted the fieldwork. LAG, PW and MPD collected the DT data. LAG, PW, MPD and MAG photographed the fossils. LAG reviewed and photographed herbarium sheets. LAG and MPD gathered extant host association records. LAG analyzed the data and wrote the manuscript with comments from all other authors.

## Disclaimer

The New Phytologist Foundation remains neutral with regard to jurisdictional claims in maps and in any institutional affiliations.

## Supporting information


**Dataset S1** Raw abundances of insect‐mediated and pathogenic damage traces in *Eucalyptus frenguelliana* leaves from the early Eocene Laguna del Hunco site.
**Dataset S2** Catalog of *Eucalyptus frenguelliana* fossil specimens and associated damage types.
**Dataset S3** Extant, rainforest‐associated *Eucalyptus* species surveyed for analog damage types comparable with the fossil specimens.
**Dataset S4** Unique (insect herbivore‐*Eucalyptus* host species) associations recorded from relevant entomological, ecological, and forestry literature.

**Fig. S1** Accumulation curves of mining damage types per number of herbarium sheets reviewed, colored by *Eucalyptus* subgenera surveyed.
**Notes S1** *Eucalyptus* herbarium specimens with damage types matching those observed in *Eucalyptus frenguelliana*.
**Notes S2** References for Dataset S4. Insect herbivores associated with *Eucalyptus*.
**Table S1** Insect herbivory damage types in fossil *Eucalyptus frenguelliana* leaves from the early Eocene Laguna del Hunco locality and extant *Eucalyptus* species with the same damage types.
Please note: Wiley is not responsible for the content or functionality of any Supporting Information supplied by the authors. Any queries (other than missing material) should be directed to the *New Phytologist* Central Office.

## Data Availability

The data that support the findings of this study are available in the Supporting Information and Datasets S1–S4. Photographs of the fossil specimens are available open access in FigShare (doi: 10.6084/m9.figshare.24756975).
