# Supplementary material for: Fossil insect‐feeding traces indicate unrecognized evolutionary history and biodiversity on Australia's iconic Eucalyptus
Source: New Phytol. 2024 Nov 28;245(4):1762–73. doi: 10.1111/nph.20316 (PMC11754931; doi:10.1111/nph.20316)
Supplement: Supplementary file 2 — Fig. S1 Accumulation curves of mining damage types per number of herbarium sheets reviewed, colored by Eucalyptus subgenera surveyed. Notes S1 Eucalyptus herbarium specimens with damage types matching those observed in Eucalyptus frenguelliana. Notes S2 References for Dataset S4. Insect herbivores associated with Eucalyptus. Table S1 Insect herbivory damage types in fossil Eucalyptus frenguelliana leaves from the early Eocene Laguna del Hunco locality and extant Eucalyptus species with the same damage types. Please note: Wiley is not responsible for the content or functionality of any Supporting Information supplied by the authors. Any queries (other than missing material) should be directed to the New Phytologist Central Office. [file NPH-245-1762-s001.docx]

***New Phytologist* Supporting Information**

**Article title:** Fossil insect-feeding traces indicate unrecognized evolutionary history on Australia’s iconic *Eucalyptus*

**Authors:** L. Alejandro Giraldo, Peter Wilf, Michael P. Donovan, Robert M. Kooyman, María A. Gandolfo.

**Article acceptance date:** 12 November 2024

The following Supporting Information is available for this article (in order of appearance):

**Notes S1** *Eucalyptus* herbarium specimens with damage types (DTs) matching those observed in *Eucalyptus frenguelliana*

**Fig. S1** Accumulation curves of mining damage types (DTs) per number of herbarium sheets reviewed, colored by *Eucalyptus* subgenera surveyed.

**Table S1** Insect herbivory damage types (DTs) in fossil *Eucalyptus frenguelliana* leaves from the early Eocene Laguna del Hunco (LH) locality and extant *Eucalyptus* species with the same damage types.

**Notes S2** References for Supporting Dataset S4. Insect herbivores associated with *Eucalyptus*.

Notes S1. *Eucalyptus* herbarium specimens with damage types (DTs) matching those observed in *Eucalyptus frenguelliana*.

We list herbarium specimens that have DTs matching those observed in the fossil specimens. For mining associations, which are emphasized in the main text for their potential to reveal ancient *Eucalyptus* associations, we provide additional geospatial information from the specimen annotations to facilitate discovery. Because more than 10,000 herbarium specimens were reviewed, it was not feasible to score all herbarium sheets for all DTs, and many of the indicated sheets have additional DTs, particularly hole and margin feeding DTs.

We report herbarium sheets from the Harvard University Herbaria, Cambridge (A and GH); the Australian National Herbarium, Canberra (CANB and CBG); the Queensland Herbarium and Biodiversity Science, Brisbane (BRI); the National Herbarium of New South Wales of the Royal Botanic Gardens and Domain Trust, Mount Annan (NSW); the Royal Botanic Gardens Victoria, Melbourne (MEL); the Naturalis Biodiversity Center, Leiden (L, U, WAG, and AMD); the Royal Botanic Garden Edinburgh (E); the Royal Botanic Gardens Kew, Richmond (K); the Muséum National d'Histoire Naturelle, Paris (P); the United States National Herbarium of the Smithsonian Institution, Washington D. C. (US); and The New York Botanical Garden, New York (NY). We report catalogue numbers exactly as they should be searched in their respective online platforms, and thus format is variable (e.g., CBG 35770.1, L.2504894, NSW314312).

**MINING**

**DT422**

**Figured**

*E. tereticornis* (CBG 35770.1; Fig 2b).

Splitter’s Creek, W of Bundaberg, on Gin Gin Road. Queensland, Australia. Collected in 1970. Lat -24.855556, Long 152.277778.

**Not figured**

*E. moluccana* (BRI AQ0130282)

Killarney. Queensland, Australia. Collected in 1917. Lat -28.341667, Long 152.291667.

**DT41**

**Figured**

*E. cloeziana* (BRI AQ0097039; Fig 2d)

Mareeba. Queensland, Australia. *Collection date not provided*. Lat -17.081801, Long 145.417777.

**Not Figured**

*E. microcorys* (A C.T.White No. 1220)

K’gari (provided as “Fraser Island” in the herbarium specimen). Queensland, Australia. Collected in 1921. *No coordinates provided*.

*E. punctata* (CANB 17112.1)

Owens Gap, Scone. New South Wales, Australia. Collected in 2007. Lat -32.05, Long 150.7.

**DT90**

**Figured**

*E. major* (CANB 446889; Fig 2t)

Maranoa district: Gt Dividing Range; ca 80 km SW of Rolleston, Kenniff Lookout. Queensland, Australia. Collected in 1977. Lat -24.901389, Long 148.

**Not figured**

*E. acmenoides* (BRI AQ0636111)

Timber Reserve 202, Many Peaks Range, near hang glider jump point. Queensland, Australia. Collected in 1995. Lat -24.37342, Long 151.459401.

*E. major* (CANB 446889)

Maranoa district: Gt Dividing Range; ca 80 km SW of Rolleston,Kenniff Lookout. Queensland, Australia. Collected in 1977. Lat -24.901389, Long 148.

*E. moluccana* (CANB 417077)

Nowra. New South Wales, Australia. Collected in 1932. Lat -34.884722, Long 150.6.

*E. platyphylla* (BRI AQ0095472)

Mt Simon Site 8E 28.8K S of Cooktown. Queensland, Australia. Collected in 1974. Lat -15.655278, Long 145.215.

**DT92**

**Figured**

*E. major* (BRI AQ0132184; Fig. 2n)

Top of Mt Coot-tha (Brisbane). Queensland, Australia. Collected in 1966. Lat -27.473406, Long 152.959402.

**Not figured**

*E. resinifera* (CANB 891861.2)

Herberton Range, between Atherton and Herberton. Queensland, Australia. Collected in 1971. Lat -17.4, Long 145.4.

**DT94**

**Figured**

*E. decolor* (BRI AQ0503557; Fig. 2f)

12.5km along road to Coongara Falls off Biggenden-Ban Ban Springs Road. Queensland, Australia. Collected in 1989. Lat -25.656667, Long 152.004167.

**Not figured**

*E. microcorys* (CANB 450112)

Coopernook State Forest. New South Wales, Australia. Collected in 1938. Lat -31.816667, Long 152.6.

**DT139**

**Figured**

*E. melliodora* (BRI AQ0130148; Fig. 2j)

Fletcher. Queensland, Australia. Collected in 1933. Lat -28.748411, Long 151.917767.

**Not figured**

*E. acmenoides* (CANB 425802)

North Kennedy. Mount Burrumbush, Bowling Green Bay National Park,S of Townsville. Queensland, Australia. Collected in 1991. Lat -19.433333, Long 147.083333.

*E. cloeziana* (CANB 123602)

32 miles W of Theodore Township. Queensland, Australia. Collected in 1963. Lat -24.951389, Long 149.65.

*E. crebra* (BRI AQ0097452)

15 miles west of Bauhinia Downs. Queensland, Australia. Collected in 1970. Lat -24.840085, Long 149.142769.

*E. dunnii* (CBG 9504045)

NW of Bonalbo, Yabbra State Forest, Section E Road. New South Wales, Australia. Collected in 1991. Lat -28.568056, Long 152.5.

*E. fibrosa* (BRI AQ0786999)

Near Prout Rd, Burbank, 14km ESE of Brisbane CBD. Queensland, Australia. Collected in 2008. Lat -27.530346, Long 153.146899.

*E. melliodora* (BRI AQ0423978)

10K E of Texas. Queensland, Australia. Collected in 1984. Lat -28.856747, Long 151.276108.

*E. nobilis* (CANB 725560)

Parson's property / Nature Refuge. Mailman Road off North Branch Road, Maryvale. Queensland, Australia. Collected in 2009. Lat -28.015278, Long 152.289444.

**DT171**

**Figured**

*E. cloeziana* (CANB 413235; 2d)

4km S of Helenvale on Rossville road. Queensland, Australia. Collected in 1973. Lat -15.75, Long 145.233333.

**Not figured**

*E. cloeziana* (BRI AQ0097055)

Warrego River Head, Ca. 100 miles NE of Augathella. Queensland, Australia. Collected in 1949. Lat -24.915106, Long 147.4178.

*E. saligna* (BRI AQ0132681)

Binna Burra. New South Wales, Australia. Collected in 1954. Lat -28.248401, Long 153.25107.

**DT185**

**Figured**

*E. crebra* (BRI AQ0491261; Fig 2l)

8.1km N of Berajondo, towards Agnes Water. Queensland, Australia. Collected in 2000. Lat -24.557029, Long 151.821063.

**DT207**

**Figured**

*E. punctata* (BRI AQ0132345; Fig 2p)

Hungry Hill, north of Gloucester. New South Wales, Australia. Collected in 1966. Lat -31.606751, Long 151.726118.

**Not figured**

*E. oreades* (BRI AQ0131070)

Blackheath. New South Wales, Australia. Collected in 1905. Lat -33.581767, Long 150.251167.

*E. punctata* (CANB 456662)

Ridge above Pearl Beach. New South Wales, Australia. Collected in 1966. Lat -33.533333, Long 151.316667.

*E. tindaliae* (BRI AQ0727591)

Blue Gum paddock, 'Tandora', c. 15km NE of Maryborough. Queensland, Australia. Collected in 2006. Lat -25.447381, Long 152.793522.

**DT210**

**Figured**

*E. robusta* (CANB 455708; Fig. 2r)

Nambucca SF, Nambucca Heads. New South Wales, Australia. Collected in 1969. Lat - 30.633333, Long 152.966667.

**Not figured**

*E. grandis* (CANB 135660.1)

Bruxner Park Reserve, ca 6mls NW of Coffs Harbour. New South Wales, Australia. Collected in 1964. Lat -30.266667, Long 153.083333.

*E. resinífera* (BRI AQ0657633)

Ridge to Coronation Lookout; Grid Ref 8063-735838. Queensland, Australia. Collected in 1995. Lat -17.325666, Long 145.811138.

*E. robusta* (CANB 455769)

Upper Stony Creek, Bowenia State Forest, N of Rockhampton. Queensland, Australia. Collected in 1978. Lat -22.866667, Long 150.633333.

**HOLE FEEDING**

**DT1**

**Figured**

*E. resinifera* (CANB 416146; Fig. 3b)

**Not figured**

*E. acmenoides* (BRI AQ0470525, BRI AQ0772134, BRI AQ0735687)

*E. andrewsii* (L.2505920)

*E. cloeziana* (BRI AQ0345654, BRI AQ0601122, BRI AQ0005551, L.2511645)

*E. crebra* (BRI AQ0470517)

*E. decolor* (BRI AQ0664777)

*E. deglupta* (L.2504894, L.2504868, L.2504824)

*E. dunnii* (NSW313319)

*E. fibrosa* (L.2506874, L.2506876)

*E. fusiformis* (BRI AQ0436158)

*E. globoidea* (L.3918809)

*E. grandis* (L.2506665)

*E. hallii* (MEL 1057326A)

*E. houseana* (NSW345270)

*E. laevopinea* (L.2506549)

*E. major* (CANB 668871.1)

*E. melliodora* (BRI AQ0670846, BRI AQ0130160)

*E. michaeliana* (BRI AQ0458700)

*E. microcorys* (BRI AQ0863625, BRI AQ0130309)

*E. moluccana* (L.2506430, L.2506428)

*E. nobilis* (L.2506352)

*E. notabilis* (L.2506377)

*E. obliqua* (L.2506338)

*E. oreades* (L.2506249)

*E. pellita* (L.2506926)

*E. pilularis* (BRI AQ0131688, L.2507265)

*E. platyphylla* (L.2507215, L.2507212)

*E. propinqua* (L.4148135)

*E. punctata* (U.1438678)

*E. raveretiana* (L.2512454)

*E. robusta* (GH J.L. Boorman 77155)

*E. rummeryi* (MEL 1612466A)

*E. saligna* (GH M.S. Clemens June 1945)

*E. siderophloia* (U.1438779)

*E. tereticornis* (U.1438772)

*E. tindaliae* (BRI AQ0472834)

**DT3**

**Figured**

*E. resinifera* (CANB 416146; Fig. 3b)

**Not figured**

*E. acmenoides* (BRI AQ0470525, BRI AQ0772134, BRI AQ0921637

*E. andrewsii* (BRI AQ0096880)

*E. cloeziana* (BRI AQ0345654, BRI AQ0601122, BRI AQ0601122, BRI AQ0005551)

*E. crebra* (BRI AQ0470517, BRI AQ0098412)

*E. decolor* (BRI AQ0664777)

*E. deglupta* (L.2504894, L.2504868, L.2504824, L.2504898)

*E. dunnii* (NSW314312, NSW313318, NSW313319)

*E. fibrosa* (L.2506875, L.2506869, L.2506876)

*E. fusiformis* (BRI AQ0436158, BRI AQ0168647)

*E. globoidea* (L.2506772)

*E. grandis* (K000279532, L.2506663, L.2506665)

*E. hallii* (L.2506600)

*E. houseana* (NSW345275)

*E. laevopinea* (L.2506549)

*E. major* (CANB 668871.1)

*E. melliodora* (BRI AQ0670846, BRI AQ0142824, BRI AQ0130160)

*E. michaeliana* (BRI AQ0463716, BRI AQ0458700, BRI AQ0459236)

*E. microcorys* (BRI AQ0863625, BRI AQ0130309)

*E. moluccana* (L.2506430)

*E. nobilis* (L.2506352)

*E. notabilis* (L.2506376, L.2506378)

*E. obliqua* (L.2506338)

*E. oreades* (L.2506248)

*E. pellita* (L.2506919)

*E. pilularis* (L.2507269)

*E. platyphylla* (L.2507215, L.2507212)

*E. propinqua* (L.4148135)

*E. punctata* (U.1438677)

*E. raveretiana* (L.2512454)

*E. robusta* (GH J.L. Boorman 77155)

*E. rummeryi* (MEL 1612466A, NSW744429)

*E. saligna* (GH M.S. Clemens June 1945)

*E. siderophloia* (L.2512200)

*E. tereticornis* (L.2512676, L.2512736)

*E. tindaliae* (BRI AQ0472834)

**DT8**

**Figured**

*E. major* (CANB 668871.1; Fig. 3d)

**Not figured**

*E. acmenoides* (BRI AQ0770784)

*E. andrewsii* (L.2505920, BRI AQ0096880)

*E. cloeziana* (BRI AQ0005551, BRI AQ0005551, BRI AQ0827767)

*E. crebra* (BRI AQ0228626, BRI AQ0429549)

*E. decolor* (BRI AQ0664777)

*E. deglupta* (L.2504893)

*E. dunnii* (NSW313318, NSW313319)

*E. fibrosa* (L.3931704)

*E. fusiformis* (BRI AQ0168647)

*E. globoidea* (L.3918809)

*E. grandis* (L.2506655)

*E. hallii* (L.2506600)

*E. houseana* (NSW345275)

*E. laevopinea* (L.2506549)

*E. major* (BRI AQ0477248)

*E. melliodora* (BRI AQ0130194, BRI AQ0130160)

*E. michaeliana* (BRI AQ0463716)

*E. microcorys* (BRI AQ0863625)

*E. moluccana* (L.2506430)

*E. nobilis* (CANB 406086)

*E. nobilis* (CANB 406091)

*E. notabilis* (L.2506378)

*E. obliqua* (L.2506342)

*E. oreades* (L.2506249)

*E. pellita* (L.2506926, L.2506917)

*E. pilularis* (L.2507274)

*E. platyphylla* (L.2507221)

*E. propinqua* (L.4148135)

*E. punctata* (L.4421730)

*E. raveretiana* (L.2512454)

*E. resinífera* (WAG.1114824)

*E. robusta* (L.2512361)

*E. rummeryi* (NSW744429)

*E. saligna* (L.2512310)

*E. siderophloia* (L.2512208)

*E. tereticornis* (L.2512688)

*E. tindaliae* (BRI AQ0600860)

**MARGIN FEEDING**

**DT12**

**Figured**

*E. acmenoides* (CANB 529450; Fig. 3h)

**Not figured**

*E. acmenoides* (BRI AQ0470525, BRI AQ0770784, BRI AQ0772134, BRI AQ0095389, BRI AQ0921637, BRI AQ0735687)

*E. andrewsii* (L.2505920, BRI AQ0640818)

*E. cloeziana* (BRI AQ0482404, BRI AQ0345654, BRI AQ0552127, BRI AQ0618692, BRI AQ0601122)

*E. crebra* (BRI AQ0530381, BRI AQ0470517, BRI AQ0482420)

*E. decolor* (BRI AQ0654126)

*E. deglupta* (L.2504894, L.2504898, L.2504857)

*E. dunnii* (NSW314312, NSW313318, NSW313319)

*E. fibrosa* (L.2506874, L.2506868)

*E. fusiformis* (BRI AQ0436158, BRI AQ0168647)

*E. globoidea* (L.2506772, L.2506773)

*E. grandis* (L.2506664)

*E. hallii* (L.2506600)

*E. houseana* (NSW345275)

*E. laevopinea* (L.2506548)

*E. major* (BRI AQ0099965, BRI AQ0640604, BRI AQ0477248)

*E. melliodora* (BRI AQ0725072, BRI AQ0670846, BRI AQ0142824, BRI AQ0130160)

*E. michaeliana* (BRI AQ0463716, BRI AQ0458700)

*E. michaeliana* (CANB 435259)

*E. microcorys* (BRI AQ0134368, BRI AQ0130317, BRI AQ0863625, BRI AQ0130309)

*E. moluccana* (L.2506432)

*E. nobilis* (BRI AQ0518060, L.2506352)

*E. notabilis* (L.2506376, L.2506378)

*E. obliqua* (L.2506332, L.2506344)

*E. oreades* (L.2506248)

*E. pellita* (L.2506922)

*E. pilularis* (BRI AQ0131688, L.2507270, L.2507269)

*E. platyphylla* (L.2507215)

*E. propinqua* (L.2512543, L.2512552)

*E. punctata* (BRI AQ0838212, L.2512512)

*E. raveretiana* (L.2512454, L.2512456)

*E. resinífera* (CANB 891861.2)

*E. resinífera* (L.2512420)

*E. robusta* (L.2512362)

*E. rummeryi* (NSW19117)

*E. saligna* (L.4148139)

*E. siderophloia* (BRI AQ0174866, L.2512201)

*E. tereticornis* (E00820022, L.2512687, L.2512671)

*E. tindaliae* (BRI AQ0131588, BRI AQ0600860)

**DT13**

**Figured**

*E. crebra* (BRI AQ0446860; Fig. 3f)

**Not figured**

*E. acmenoides* (BRI AQ0772134, BRI AQ0095362, BRI AQ0095323, BRI AQ0095324)

*E. andrewsii* (L.2505920, BRI AQ0640818, BRI AQ0097039)

*E. cloeziana* (BRI AQ0482404, BRI AQ0552127, BRI AQ0193556)

*E. crebra* (BRI AQ0530381, BRI AQ0470517, BRI AQ0491257, BRI AQ0482420)

*E. decolor* (BRI AQ0654126)

*E. deglupta* (L.2504857)

*E. dunnii* (NSW314312, NSW313318)

*E. fibrosa* (L.2506874, L.2506875, L.2506869)

*E. fusiformis* (BRI AQ0436158, BRI AQ0168647)

*E. globoidea* (L.2506772, L.2506773)

*E. grandis* (K000279532, L.2506657)

*E. hallii* (L.2506600)

*E. houseana* (NSW345275)

*E. laevopinea* (L.2506548)

*E. major* (BRI AQ0519508, BRI AQ0099965, BRI AQ0640604)

*E. melliodora* (BRI AQ0142824, BRI AQ0130160)

*E. michaeliana* (BRI AQ0463716, BRI AQ0458700)

*E. microcorys* (BRI AQ0134368, BRI AQ0863625, BRI AQ0130309)

*E. microcorys* (CANB 472647.1)

*E. moluccana* (L.2506432, L.2506424)

*E. nobilis* (L.2506352)

*E. notabilis* (L.2506376, L.2506377)

*E. obliqua* (L.2506347)

*E. oreades* (L.2506248)

*E. pellita* (L.2506918)

*E. pilularis* (L.2507270)

*E. platyphylla* (L.2507229)

*E. propinqua* (L.2512542)

*E. punctata* (L.2512503)

*E. raveretiana* (L.2512454, L.2512456)

*E. resinífera* (L.2512426)

*E. robusta* (L.2512362)

*E. rummeryi* (NSW19117, MEL 1612466A)

*E. saligna* (AMD.789228, L.2512316)

*E. siderophloia* (L.2512201)

*E. tereticornis* (E00820022, L.2512687, L.2512671)

*E. tindaliae* (BRI AQ0472834)

**DT14**

**Figured**

*E. acmenoides* (CANB 529450; Fig. 3h)

**Not figured**

*E. acmenoides* (BRI AQ0470525, BRI AQ0095389, BRI AQ0000415, BRI AQ0095324)

*E. andrewsii* (L.2505920, BRI AQ0640818)

*E. cloeziana* (BRI AQ0482404, BRI AQ0552127, BRI AQ0618692, BRI AQ0601122)

*E. crebra* (BRI AQ0530381, BRI AQ0491261, BRI AQ0098412, BRI AQ0482420)

*E. decolor* (BRI AQ0654126)

*E. deglupta* (L.2504894, L.2504876, L.2504857)

*E. dunnii* (NSW313319)

*E. fibrosa* (L.2506874, L.2506875, L.2506868)

*E. fusiformis* (BRI AQ0436158, BRI AQ0168647)

*E. globoidea* (L.2506773)

*E. grandis* (K000279532, L.2506673, L.2506664)

*E. hallii* (MEL 1057326A)

*E. houseana* (NSW345275)

*E. laevopinea* (L.2506548)

*E. major* (BRI AQ0099965, BRI AQ0640604)

*E. melliodora* (BRI AQ0670846, BRI AQ0130160)

*E. michaeliana* (CANB 435263, BRI AQ0463716, BRI AQ0458700, BRI AQ0459236)

*E. microcorys* (BRI AQ0134368, BRI AQ0863625)

*E. moluccana* (L.2506424)

*E. nobilis* (CANB 406105)

*E. notabilis* (L.2506377, L.2506378)

*E. obliqua* (L.2506342)

*E. oreades* (L.2506248)

*E. pellita* (L.2506922, L.3917720)

*E. pilularis* (L.2507269, L.2507272)

*E. platyphylla* (L.2507212, L.2507214)

*E. propinqua* (L.2512543, L.2512552)

*E. punctata* (BRI AQ0132347, L.2512512, L.2512503)

*E. raveretiana* (L.2512454, L.391879, L.2512456)

*E. resinífera* (L.2512420)

*E. robusta* (L.2512362, L.2512404)

*E. rummeryi* (NSW19117)

*E. saligna* (AMD.789228, L.2512316, L.4148139)

*E. siderophloia* (BRI AQ0174866)

*E. tereticornis* (E00820022, L.2512687)

*E. tindaliae* (BRI AQ0131588, BRI AQ0600860)

**DT15**

**Figured**

*E. fibrosa* (GH L.A.S. Johnson 61233; Fig. 3j)

**Not figured**

*E. acmenoides* (BRI AQ0470525, BRI AQ0000415)

*E. andrewsii* (L.2505920)

*E. cloeziana* (BRI AQ0345654, BRI AQ0552127, BRI AQ0601122, BRI AQ0601122)

*E. crebra* (BRI AQ0098412, BRI AQ0097310, BRI AQ0482420)

*E. decolor* (BRI AQ0664775)

*E. deglupta* (L.2504876, L.2504868, L.2504898)

*E. dunnii* (NSW313318)

*E. fibrosa* (L.2506869, L.2506877)

*E. fusiformis* (BRI AQ0168647)

*E. globoidea* (L.2506772, L.3918809)

*E. grandis* (L.3931714)

*E. hallii* (MEL 1057326A)

*E. houseana* (NSW345270)

*E. laevopinea* (L.2506548)

*E. major* (BRI AQ0099965, BRI AQ0640604)

*E. melliodora* (BRI AQ0670846, BRI AQ0142824, BRI AQ0130160)

*E. michaeliana* (BRI AQ0463716, BRI AQ0459236)

*E. microcorys* (BRI AQ0134368, BRI AQ0130317, BRI AQ0863625, BRI AQ0130309)

*E. moluccana* (L.2506426)

*E. nobilis* (L.2506353)

*E. notabilis* (L.2506378)

*E. obliqua* (U.1438845)

*E. oreades* (L.2506249)

*E. pellita* (L.2506922)

*E. pilularis* (U.1438799)

*E. platyphylla* (L.2507217)

*E. propinqua* (L.2512542, L.2512544)

*E. punctata* (L.2512506)

*E. raveretiana* (L.2512454)

*E. resinífera* (L.2512426)

*E*. *robusta* (L.2512361)

*E. rummeryi* (NSW744429)

*E. saligna* (AMD.789228, L.4148139)

*E. siderophloia* (BRI AQ0146076, U.1438779)

*E. tereticornis* (E00820022)

*E. tindaliae* (BRI AQ0472834)

**DT81**

**Figured**

*E. grandis* (CANB 699345; Fig. 3l)

**Not figured**

*E. michaeliana* (CANB 435263)

**SURFACE FEEDING**

**DT30**

**Figured**

*E. fibrosa* (CANB 409185; Fig. 3p)

**Not figured**

*E. crebra* (BRI AQ0465409)

*E. fibrosa* (CANB 400100)

*E. robusta* (CANB 15881)

**DT31**

**Figured**

*E. robusta* (CANB 15881; Fig. 3n)

**Not figured**

*E. tereticornis* (A R. Pullen No. 7221)

**SKELETONIZATION**

**DT16**

**Figured**

*E. michaeliana* (CANB 435213; Fig. 3r)

**Not figured**

*E. acmenoides* (BRI AQ0095323)

*E. cloeziana* (L.2511637)

*E. melliodora* (BRI AQ0130160)

*E. robusta* (A M. R. Jacobs 99310)

*E. tereticornis* (A R. Pullen No. 7221)

**DT17**

**Figured**

*E. tereticornis* (CANB 120052.1; Fig. 3t)

**Not figured**

*E. acmenoides* (BRI AQ0921637; BRI AQ0095338)

*E. crebra* (BRI AQ0098408)

*E. resinífera* (A C. T. White No. 1827)

**PIERCING-AND-SUCKING**

**DT77**

**Figured**

*E. notabilis* (CBG 295.1; Fig. 3v)

**GALLING**

**DT32**

**Figured**

*E. punctata* (BRI AQ0838212; Fig. 3x)

**Not figured**

*E. acmenoides* (BRI AQ0921637, BRI AQ0921637, BRI AQ0095353)

*E. andrewsii* (A J. L. Boorman 62235)

*E. cloeziana* (BRI AQ0097044)

*E. crebra* (BRI AQ0532460)

*E. melliodora* (BRI AQ0418032, BRI AQ0000573)

*E. propinqua* (A C. T. White No. 7791)

**DT33**

**Figured**

*E. propinqua* (A C. T. White No. 7791; Fig. 3y)

**Not figured**

*E. acmenoides* (BRI AQ0921637)

*E. crebra* (BRI AQ0134361, BRI AQ0446860, BRI AQ0532460)

**DT34**

**Figured**

*E. punctata* (BRI AQ0838212; Fig. 3x)

**Not figured**

*E. andrewsii* (A J. L. Boorman 62235)

*E. punctata* (CANB 457486)

**DT49**

**Figured**

*E. punctata* (CBG 7805142.1; Fig. 3aa)

**Not figured**

*E. crebra* (BRI AQ0509090)

*E. michaeliana* (CANB 435259)

*E. moluccana* (CANB 459853)

**DT85**

**Figured**

E. tereticornis (CANB 51504.1; Fig. 3cc)

**Not figured**

*E. crebra* (BRI AQ0639236, BRI AQ0446860, BRI AQ0532460, BRI AQ0717704, BRI AQ0717704)

*E. dunnii* (BRI AQ0098465)

*E. fibrosa* (BRI AQ0132911)

*E. laevopinea* (BRI AQ0405377)

*E. nobilis* (CBG 7702033.1, BRI AQ0518060)

E. nobilis (CBG 7702033.1)

*E. propinqua* (CANB 457303)

*E. resinifera* (CBG 150.1)

*E. siderophloia* (BRI AQ0174866)

*E. tereticornis* (BRI AQ0133386, CANB 739611.2)

E. tereticornis (CANB 726772)

**Figure S1**


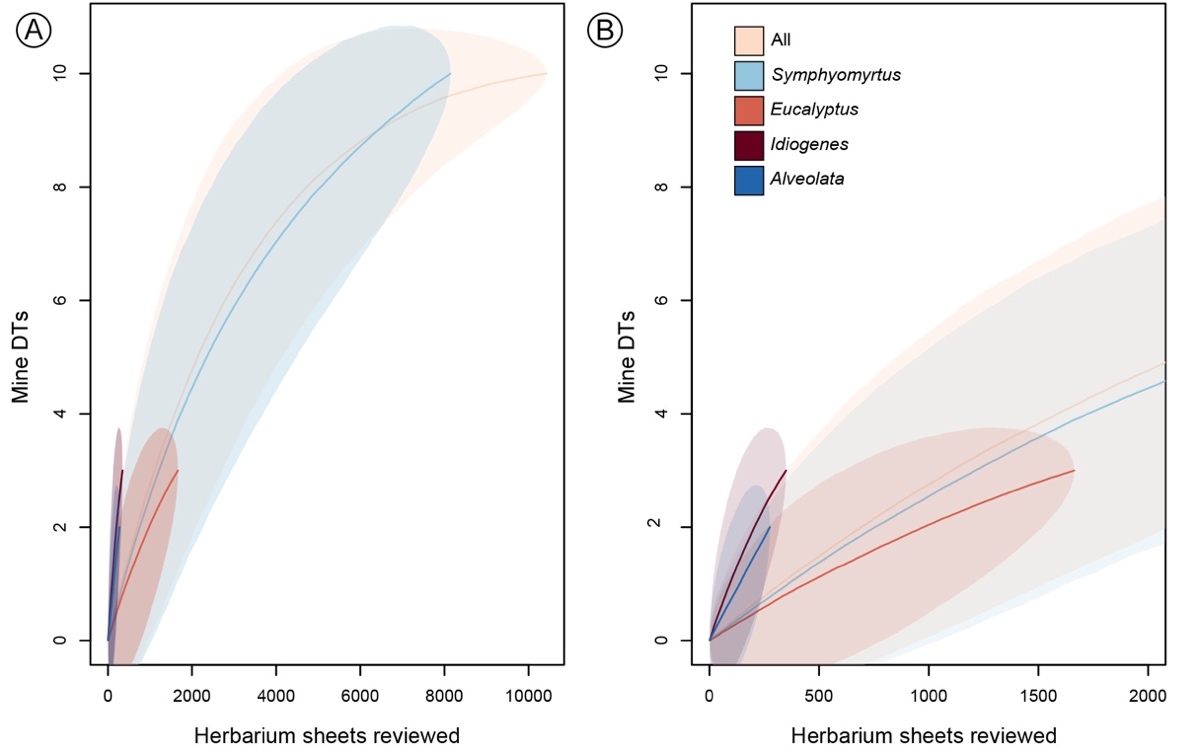


**Figure S1.** Accumulation curves of mining damage types (DTs) per number of herbarium sheets reviewed, colored by *Eucalyptus* subgenera surveyed (see Materials and Methods). Analyses were performed in R Statistical Software (v4.1.1; R Core Team, 2021) using the *vegan* package (Oksanen *et al.*, 2022). Solid lines are means of mining DTs accumulation curves, resampled without replacement for 5000 iterations; shaded areas indicate 95% confidence intervals. Panel B shows the same data as Panel A, cropped to 2000 sheets for improved visualization.

Table S1. Insect herbivory damage types (DTs) in fossil *Eucalyptus frenguelliana* leaves from the early Eocene Laguna del Hunco (LH) locality and extant *Eucalyptus* species with the same damage types (see Supporting Dataset S2 and S3). Asterisks indicate DTs known as fossils only in *E. frenguelliana* at LH. Occurrence raw totals for fossils only, out of 284 total specimens housed at MEF. Census data include field tallies made in 1999 and 2002 (see Wilf *et al.*, 2005) in which all specimens (with or without insect damage) were assessed. This represents a quantitative subset that remains a majority of the total collection through 2023, for which all specimens with insect damage (but not all those without) have been collected. Museum codes for the illustrated fossils (MPEF-Pb) are given in the Figure column.

| **Insect damage** | **DT** | **Occurrence (total)^a^** | **Occurrence (census)^b^** | **Figure** | **LH locality** | **Extant *Eucalyptus* species with analog damage, by subgenus** |
| --- | --- | --- | --- | --- | --- | --- |
| **Mining** |  |  |  |  |  |  |
| Linear, alongside midvein | 422* | 1 | 0 | Fig. 2a, b  a MPEF-Pb 13038 | 13 | ***Symphyomyrtus***  *E. tereticornis, E. moluccana* |
| Serpentine, threadlike, minimum width increase | 41 | 1 | 1 | Fig. 2g, h  g MPEF-Pb 2358 | 13 | ***Alveolata***  *E. microcorys*  ***Idiogenes***  *E. cloeziana*  ***Symphyomyrtus***  *E. punctata* |
| Linear, blunt margins, central frass trail | 90 | 1 | 1 | Fig. 2s, t  s MPEF-Pb 2229 | 13 | ***Eucalyptus***  *E. acmenoides*  ***Symphyomyrtus***  *E. major, E. moluccana, E. platyphylla* |
| Serpentine, highly coiled, solid frass | 92 | 1 | 1 | Fig. 2m, n  m MPEF-Pb 2347 | 13 | ***Symphyomyrtus***  *E. major, E. resinifera* |
| Serpentine, border variably developed | 94* | 1 | 0 | Fig. 2e, f  e MPEF-Pb 13039 | 6 | ***Alveolata***  *E. microcorys*  ***Symphyomyrtus***  *E. decolor* |
| Linear, deeply embedded, border thick | 139* | 3 | 3 | Fig. 2i, j  i MPEF-Pb 2323 | 13, 23 | ***Eucalyptus***  *E. acmenoides*  ***Idiogenes***  *E. cloeziana*  ***Symphyomyrtus***  *E. crebra, E. dunnii, E. fibrosa, E. melliodora, E. nobilis* |
| Serpentine, frass-filled, elliptical terminal chamber | 171* | 2 | 1 | Fig. 2c, d  c MPEF-Pb 2247 | 13 | ***Idiogenes***  *E. cloeziana*  ***Symphyomyrtus***  *E. saligna* |
| Curvilinear, massive reaction rim | 185* | 1 | 1 | Fig. 2k, l  k MPEF-Pb 2243 | 13 | ***Symphyomyrtus***  *E. crebra* |
| Bifurcated, round borders, frass on side | 207* | 1 | 1 | Fig. 2o, p  o MPEF-Pb 13041 | 17 | ***Eucalyptus***  *E. oreades, E. tindaliae*  ***Symphyomyrtus***  *E. punctata* |
| Short, thin, threadlike, bounded by secondary veins | 210* | 1 | 1 | Fig. 2q, r  q MPEF-Pb 2321 | 22 | ***Symphyomyrtus***  *E. grandis, E. resinifera, E. robusta* |
| **Hole feeding** |  |  |  |  |  |  |
| Circular, <1 mm in diameter | 1 | 16 | 16 | Fig. 3a, b  a MPEF-Pb 2338 | 4, 6, 13, 15 | ***Alveolata***  *E. microcorys*  ***Eucalyptus***  *E. acmenoides, E. andrewsii, E. globoidea, E. laevopinea, E. obliqua, E. oreades, E. pilularis, E. tindaliae*  ***Idiogenes***  *E. cloeziana*  ***Symphyomyrtus***  *E. crebra, E. decolor, E. deglupta, E. dunnii, E. fibrosa, E. fusiformis, E. grandis, E. hallii, E. houseana, E. major, E. melliodora, E. michaeliana, E. moluccana, E. nobilis, E. notabilis, E. pellita, E. platyphylla, E. propinqua, E. punctata, E. raveretiana, E. resinífera, E. robusta, E. rummeryi, E. saligna, E. siderophloia, E. tereticornis* |
| Polylobate, 1–5 mm in diameter | 3 | 43 | 39 | Fig. 3a, b  a MPEF-Pb 2338 | 2, 3, 6, 13, 22, 27 |  |
| Parallel sided slots | 8 | 2 | 2 | Fig. 3c, d  c MPEF-Pb 2245 | 6, 13 |  |
| **Margin feeding** |  |  |  |  |  |  |
| Arcuate excision | 12 | 65 | 48 | Fig. 3g, h  g MPEF-Pb 13033 | 2, 4, 6, 13, 15, 22, 25, 27, 29, 30 |  |
| Leaf apex excision | 13 | 17 | 9 | Fig. 3e, f  e MPEF-Pb 13030 | 2, 4, 6, 13, 15, 16, 25, 27, 30 |  |
| Excision reaching midvein | 14 | 38 | 30 | Fig. 3g, h  g MPEF-Pb 13033 | 2, 4, 6, 13, 15, 25, 27 |  |
| Deeply trenched excision | 15 | 3 | 3 | Fig. 3i, j  i MPEF-Pb 2322 | 6, 13 | ***Alveolata***  *E. microcorys*  ***Eucalyptus***  *E. acmenoides*  ***Idiogenes***  *E. cloeziana*  ***Symphyomyrtus***  *E. crebra, E. fibrosa, E. melliodora* |
| Nearly perfect circular excision | 81 | 2 | 1 | Fig. 3k, l  k MPEF-Pb 13032 | 6, 27 | ***Symphyomyrtus***  *E. grandis, E. michaeliana* |
| **Surface feeding** |  |  |  |  |  |  |
| Polylobate abrasion, strong reaction rim | 30 | 7 | 5 | Fig. 3o, p  o MPEF-Pb 2250 | 13 | ***Symphyomyrtus***  *E. crebra, E. fibrosa, E. robusta* |
| Circular–elliptical abrasion, strong reaction rim | 31 | 8 | 4 | Fig. 3m, n  m MPEF-Pb 2261 | 4, 13, 27 | ***Symphyomyrtus***  *E. robusta, E. tereticornis* |
| **Skeletonization** |  |  |  |  |  |  |
| Interveinal tissue removed, no reaction rim | 16 | 12 | 10 | Fig. 3q, r  q MPEF-Pb 13034 | 4, 6, 13, 15, 22, 25 | ***Eucalyptus***  *E. acmenoides*  ***Idiogenes***  *E. cloeziana*  ***Symphyomyrtus***  *E. melliodora, E. michaeliana, E. robusta, E. tereticornis* |
| Interveinal tissue removed, reaction rim | 17 | 25 | 15 | Fig. 3s, t  s MPEF-Pb 2327 | 2, 4, 6, 13, 15, 25, 27 | ***Eucalyptus***  *E. acmenoides*  ***Symphyomyrtus***  *E. crebra, E. resinífera, E. tereticornis* |
| **Piercing-and-sucking** |  |  |  |  |  |  |
| Scale insect covers, clustered | 77* | 2 | 2 | Fig. 3u, v  u MPEF-Pb 2362 | 2 | ***Symphyomyrtus***  *E. notabilis* |
| **Galling** |  |  |  |  |  |  |
| Dark, circular, in interveinal tissue | 32 | 42 | 22 | Fig. 3w, x  w MPEF-Pb 2353 | 1, 2, 4, 6, 13, 15, 16, 22, 25, 27 | ***Eucalyptus***  *E. acmenoides, E. andrewsii*  ***Idiogenes***  *E. cloeziana*  ***Symphyomyrtus***  *E. crebra, E. melliodora, E. platyphylla, E. punctata* |
| Dark, circular, along primary veins | 33 | 21 | 16 | Fig. 3w, y  w MPEF-Pb 2353 | 4, 6, 13, 16, 17, 25, 27 | ***Eucalyptus***  *E. acmenoides*  ***Symphyomyrtus***  *E. crebra, E. propinqua* |
| Dark, circular, along secondary veins | 34 | 3 | 3 | Fig. 3w, x  w MPEF-Pb 2353 | 4, 13 | ***Eucalyptus***  *E. andrewsii, E. pilularis*  ***Symphyomyrtus***  *E punctata* |
| Single, flat, carbonized core and wall | 49* | 6 | 3 | Fig. 3z, aa  z MPEF-Pb 2283 | 4, 6, 13, 28 | ***Symphyomyrtus***  *E. crebra, E. michaeliana, E. moluccana, E. punctata* |
| Single, lenticular, along primary vein | 85 | 2 | 2 | Fig. 3bb, cc  bb MPEF-Pb 2251 | 6, 13 | ***Eucalyptus***  *E. laevopinea*  ***Symphyomyrtus***  *E. crebra, E. dunnii, E. fibrosa, E. nobilis, E. propinqua, E. resinífera, E. siderophloia, E. tereticornis* |

^a^Total number of leaves collected and housed at MEF adds to 284 leaves (189 damaged, 95 undamaged).

^b^Total number of censused leaves adds to 508 leaves (137 damaged, 371 undamaged).

**Supporting Information (SI) References**

**Nicolle D**. **2015**. *Classification of the eucalypts (Angophora*, *Corymbia and Eucalyptus). Version 2*. Available at: http://www.dn.com.au/Classification-of-the-Eucalypts.html

**Oksanen J, Simpson GL, Blanchet G, Kindt R, Legendre P, Minchin PR, O’Hara RB, Solymos P, Stevens MHH, Szoecs E, *et al.*** **2022**. vegan: Community Ecology Package.

**R Core Team**. **2021**. R: A Language and Environment for Statistical Computing.

**Thornhill AH, Crisp MD, Külheim C, Lam KE, Nelson LA, Yeates DK, Miller JT**. **2019**. A dated molecular perspective of eucalypt taxonomy, evolution and diversification. *Australian Systematic Botany* **32**: 29–48.

**Wilf P, Cúneo NR, Johnson KR, Hicks JF, Wing SL, Obradovich JD**. **2003**. High plant diversity in Eocene South America: evidence from Patagonia. *Science* **300**: 122–125.

**Wilf P, Labandeira CC, Johnson KR, Cúneo NR**. **2005**. Richness of plant–insect associations in Eocene Patagonia: a legacy for South American biodiversity. *Proceedings of the National Academy of Sciences U.S.A.* **102**: 8944–8948.

**Notes S2.** References for Supporting Dataset S4. Insect herbivores associated with *Eucalyptus*.

**Abbott I, Wills A, Burbidge T**. **1999**. Reinfestation of *Eucalyptus marginata* ground coppice by jarrah leafminer after scorch by autumn or spring fires. *Australian Forestry* **62**: 160–165.

**dos Anjos N, Majer JD, Loch AD**. **2002**. Occurrence of the eucalypt leaf beetle, *Cadmus excrementarius* Suffrian (Coleoptera: Chrysomelidae: Cryptocephalinae), in Western Australia. *Journal of the Royal Society of Western Australia* **85**: 161–164.

**Beardsley Jr. JW**. **1974a**. A new genus of Coccoidea from Australian *Eucalyptus* (Homoptera). *Proceedings of the Hawaiian Entomological Society* **21**: 325–328.

**Beardsley Jr. JW**. **1974b**. A review of the genus *Sphaerococcopsis* Cockerell, with descriptions of two new species (Homoptera: Coccoidea). *Proceedings of the Hawaiian Entomological Society* **21**: 329–342.

**Ben-Dov Y**. **1994**. *A Systematic Catalogue of the Mealybugs of the World (Insecta: Homoptera: Coccoidea: Pseudococcidae and Putoidae) with Data on Geographical Distribution, Host Plants, Biology and Economic Importance*. Andover, Netherlands: Intercept Limited.

**Berry JA, Withers TM**. **2002**. New gall-inducing species of ormocerine pteromalid (Hymenoptera: Pteromalidae: Ormocerinae) described from New Zealand. *Australian Journal of Entomology* **41**: 18–22.

**Bhatti S, Gullan PJ**. **1990**. New margarodid species (Homoptera: Coccoidea: Margarodidae: Monophlebinae) from New Guinea. *Invertebrate Taxonomy* **3**: 877–911.

**Blackburn T**. **1897**. Revision of the genus *Paropsis*. Part I. *Proceedings of the Linnean Society of New South Wales.* **21**: 637–693.

**Borchsenius NS**. **1966**. *A Catalogue of the Armoured Scale Insects (Diaspidoidea) of the World*. Moscow, Russia: Nauka.

**Borowiec N, Salle JL, Brancaccio L, Thaon M, Warot S, Branco M, Ris N, Malausa J-C, Burks R**. **2019**. *Ophelimus mediterraneus* sp. n. (Hymenoptera, Eulophidae): a new *Eucalyptus* gall wasp in the Mediterranean region. *Bulletin of Entomological Research* **109**: 678–694.

**Brimblecombe AR**. **1953**. Studies of the Coccoidea. 1. New species of *Neoleonardia*. *Queensland Journal of Agricultural Science* **10**: 161–166.

**Brimblecombe AR**. **1955**. Studies of the Coccoidea. 3. The genera *Chentraspis*, *Clavaspis*, *Lindingaspis* and *Morganella* in Queensland. *Queensland Journal of Agricultural Science* **12**: 39–56.

**Brimblecombe AR**. **1956**. Studies of the Coccoidea. 5. The genus *Ceroplastes* in Queensland. *Queensland Journal of Agricultural Science* **13**: 159–167.

**Brimblecombe AR**. **1957**. Studies of the Coccoidea. 6. New genera and new species of Aspidiotini. *Queensland Journal of Agricultural Science* **14**: 261–291.

**Brimblecombe AR**. **1958**. Studies of the Coccoidea. 7. New designations of some Australian Diaspididae. *Queensland Journal of Agricultural Science* **15**: 59–94.

**Brimblecombe AR**. **1959**. Studies of the Coccoidea. 8. Three new genera and sixteen new species of Aspidiotini. *Queensland Journal of Agricultural Science* **16**: 121–156.

**Brimblecombe AR**. **1962**. Studies of the Coccoidea. 13. The genera *Aonidiella*, *Chrysomphalus* and *Quadraspidiotus* in Queensland. *Queensland Journal of Agricultural Science* **19**: 403–423.

**Brimblecombe AR**. **1968**. Studies of the Coccoidea. 14. The genera *Aspidiotus*, *Diaspidiotus* and *Hemiberlesia* in Queensland. *Queensland Journal of Agricultural and Animal Sciences* **25**: 39–56.

**Brown SW**. **1967**. Chromosome systems of the Eriococcidae (Coccoidea-Homoptera). I. A survey of several genera. *Chromosoma* **22**: 126–150.

**Carnegie AJ**. **2008**. *Healthy Hardwoods: A Field Guide to Pests, Diseases and Nutritional Disorders in Subtropical Hardwoods*. Victoria, Australia: Forest & Wood Products Australia.

**Carter JJ, Edwards DW, Humphreys FR**. **1981**. Eucalypt diebacks in New South Wales. In: Old KM, Kile GA, Ohmart CP, eds. *Eucalypts Dieback in Forests and Woodlands*. Melbourne, Australia: CSIRO Publishing, 27–30.

**Cockerell TDA**. **1899a**. Notes on Australian Coccidae 1. *Victorian Naturalist* **1**: 13–16.

**Cockerell TDA**. **1899b**. Notes on Australian Coccidae 3. *Victorian Naturalist* **16**: 88–89.

**Common IFB**. **1981**. Some factors responsible for imbalances in the Australian fauna of Lepidoptera. *Journal of the Lepidopterists’ Society* **34**: 286–294.

**Common IFB**. **1990**. *Moths of Australia*. Melbourne, Australia: Melbourne University Publishing.

**Cook LG**. **2000**. Extraordinary and extensive karyotypic variation: A 48-fold range in chromosome number in the gall-inducing scale insect *Apiomorpha* (Hemiptera: Coccoidea: Eriococcidae. *Genome* **43**: 255–263.

**Cook LG**. **2001**. Extensive chromosomal variation associated with taxon divergence and host specificity in the gall-inducing scale insect *Apiomorpha munita* (Schrader) (Hemiptera: Sternorrhyncha: Coccoidea: Eriococcidae). *Biological Journal of the Linnean Society* **72**: 265–278.

**Cook LG**. **2003**. *Apiomorpha gullanae* sp. n., an unusual new species of gall-inducing scale insect (Hemiptera: Eriococcidae). *Australian Journal of Entomology* **42**: 327–333.

**Cook LG, Gullan PJ**. **2008**. Insect, not plant, determines gall morphology in the *Apiomorpha pharetrata* species-group (Hemiptera: Coccoidea). *Australian Journal of Entomology* **47**: 51–57.

**Cox JM**. **1987**. *Pseudococcidae (Insecta: Hemiptera). Fauna of New Zealand* (CT Duval, Ed.). Wellington, New Zealand: DSIR Science Information Publishing Centre.

**Curry SJ**. **1981**. The association of insects with eucalypt dieback in southwestern Australia. In: Old KM, Kile GA, Ohmart CP, eds. *Eucalypt Dieback in Forests and Woodlands*. Melbourne, Australia: CSIRO Publishing, 130–133.

**Dittrich-Schröder G, Hurley BP, Wingfield MJ, Nahrung HF, Slippers B**. **2020**. Invasive gall-forming wasps that threaten non-native plantation-grown *Eucalyptus*: diversity and invasion patterns. *Agricultural and Forest Entomology* **22**: 285–297.

**Edwards PB, Wanjura WJ, Brown WV**. **1993**. Selective herbivory by Christmas beetles in response to intraspecific variation in *Eucalyptus* terpenoids. *Oecologia* **95**: 551–557.

**Elliott HJ, Bashford R, Palzer C**. **1981**. Defoliation of dry sclerophyll eucalypt forest by *Stathmorrhopa aphotista* Turner (Lepidoptera: Geometridae) in southern Tasmania. In: Old KM, Kile GA, Ohmart CP, eds. *Eucalypt Dieback in Forests and Woodlands*. Melbourne, Australia: CSIRO Publishing, 134–139.

**Farr JD**. **2020**. Herbarium specimens provide historical evidence of *Cardiaspina jerramungae* (Hemiptera: Psylloidea, Aphalaridae) outbreaks on *Eucalyptus occidentalis* in the Lower Great Southern of Western Australia. *Austral Entomology* **59**: 167–177.

**Farrow R**. **2016**. *Insects of South-Eastern Australia: An Ecological and Behavioural Guide*. Melbourne, Australia: CSIRO Publishing.

**Felton K**. **1981**. *Eucalyptus* diebacks in Tasmania. In: Old KM, Kile GA, Ohmart CP, eds. *Eucalypt Dieback in Forests and Woodlands*. Melbourne, Australia: CSIRO Publishing, 51–54.

**Fernald ME**. **1903**. A catalogue of the Coccidae of the world. *Bulletin of the Hatch Experiment Station of the Massachusetts Agricultural College* **88**: 1–360.

**Flock RA**. **1957**. Biological notes on a new Chalcid-fly from seed-like *Eucalyptus* galls in California. *The Pan-Pacific Entomologist* **33**: 153–155.

**Floyd R, Wylie R, Old K, Dudzinksi M, Kile G**. **1998**. *Pest Risk Analysis of* Eucalyptus *spp. at Risk from Incursions of Plant Pests and Pathogens through Australia’s Northern Border*. Canberra, Australia: CSIRO Entomology.

**Froggatt WW**. **1893**. Notes on the family Brachyscelidae, with some account of their parasites, and descriptions of new species. Part I. *Proceedings of the Linnean Society of New South Wales* **7**: 353–378.

**Froggatt WW**. **1894a**. Notes on the family Brachyscelidae, with descriptions of new species, Part II. *Proceedings of the Linnean Society of New South Wales* **8**: 209–214.

**Froggatt WW**. **1894b**. Notes on the family Brachyscelidae, with descriptions of new species. Part III. *Proceedings of the Linnean Society of New South Wales* **8**: 335–348.

**Froggatt WW**. **1898**. Notes on the subfamily Brachyscelinae, with descriptions of new species. Part V. *Proceedings of the Linnean Society of New South Wales* **23**: 370–379.

**Froggatt WW**. **1914**. A descriptive catalogue of the scale insects (‘Coccidae’) of Australia (Part I). *Agricultural Gazette of New South Wales* **25**: 127–136.

**Froggatt WW**. **1915**. A descriptive catalogue of the scale insects ('Coccidae’) of Australia. *Agricultural Gazette of New South Wales* **26**: 411–423, 511–516, 603–615, 754–764, 1055–1064.

**Froggatt WW**. **1916**. A descriptive catalogue of the scale insects (‘Coccidae’) of Australia. (Part II). *Agricultural Gazette of New South Wales* **27**: 425–430, 568–577, 809–816.

**Froggatt WW**. **1921**. *A Descriptive Catalogue of the Scale Insects (‘Coccidae’) of Australia. (Part II)*. Sydney, Australia: W. A. Gullick Government Printer.

**Froggatt WW**. **1925**. Notes on Australian Coccidae with descriptions of new species. *Proceedings of the Linnean Society of New South Wales* **50**: 378–380.

**Froggatt WW**. **1927**. *Forest Insects and Timber Borers*. Sydney, Australia: A. J. Kent, Government Printer.

**Froggatt WW**. **1929**. Notes on gall-making coccids with descriptions of new species. *Proceedings of the Linnean Society of New South Wales* **54**: 375–378.

**Froggatt WW**. **1930**. Notes on gall-making coccids with descriptions of new species. II. *Proceedings of the Linnean Society of New South Wales* **55**: 468–474.

**Froggatt WW**. **1931**. A classification of the gall-making coccids of the genus *Apiomorpha*. *Proceedings of the Linnean Society of New South Wales* **56**: 431–454.

**Fuller C**. **1897a**. A gall-making diaspid. *Agricultural Gazette of New South Wales* **8**: 579–580.

**Fuller C**. **1897b**. Coccid literature. *Journal of Western Australia Bureau of Agriculture* **4**: 1342–1343.

**Fuller C**. **1897c**. Some Coccidae of Western Australia. *Journal of Western Australia Bureau of Agriculture* **4**: 1344–1346.

**Fuller C**. **1899**. XIV. Notes and descriptions of some species of Western Australian Coccidae. *Transactions of the Entomological Society of London* **1899**: 435–473.

**Girault AA**. **1915**. Australian Hymenoptera Chalcidoidea, XII. The family Callimomidae with descriptions of new genera and species. *Memoirs of the Queensland Museum* **4**: 275–309.

**Greaves R**. **1966**. Insect defoliation of Eucalypt regrowth in the Florentine Valley, Tasmania. *Appita* **19**: 119–126.

**Green EE**. **1900**. Descriptions of new Victorian Coccidae. *The Victorian Naturalist* **17**: 9–14.

**Green EE**. **1904**. Notes on Australian Coccidae, ex. coll. W.W. Froggatt, with descriptions of new species. No. I. *Proceedings of the Linnean Society of New South Wales* **29**: 462–465.

**Green EE**. **1905**. Some new Victorian Coccidae. *Victorian Naturalist* **22**: 3–8.

**Green EE**. **1915**. New species of Coccidae from Australia. *Bulletin of Entomological Research* **6**: 45–53.

**Green EE**. **1916**. Remarks on Coccidae from Northern Australia - II. *Bulletin of Entomological Research* **7**: 53–65.

**Green EE**. **1929**. Some Coccidae collected by Dr. J.G. Myers in New Zealand. *Bulletin of Entomological Research* **19**: 369–389.

**Grissell EE**. **2006**. A new species of *Megastigmus* Dalman (Hymenoptera : Torymidae), galling seed capsules of *Eucalyptus camaldulensis* Dehnhardt (Myrtaceae) in South Africa and Australia. *African Entomology* **14**: 87–94.

**Gullan PJ**. **1984**. A revision of the gall-forming coccoid genus *Apiomorpha* Rübsaamen (Homoptera: Eriococcidae: Apiomorphinae). *Australian Journal of Zoology, Supplementary Series* **97**: 1–203.

**Gullan PJ**. **1999**. A new genus of subcortical coccoids (Hemiptera: Coccoidea: Eriococcidae) on *Eucalyptus*. *Memoirs of Museum Victoria* **57**: 241–250.

**Gullan PJ, Davis MR, Cook LG**. **1997**. The response of gall-inducing scale insects (Hemiptera: Eriococcidae: *Apiomorpha* Rübsaamen) to the fire history of mallee eucalypts in Danggali Conservation Park, South Australia. *Transactions of the Royal Society of South Australia* **121**: 137–146.

**Gullan PJ, Jones MG**. **1989**. A new species of gall-forming coccoid (Insecta: Homoptera: Eriococcidae) from Western Australia. *Record of the Western Australian Museum* **14**: 321–329.

**Gullan PJ, Strong KL**. **1997**. Scale insects under eucalypt bark: a revision of the Australian genus *Phacelococus* Miller (Hemiptera: Eriococcidae. *Australian Journal of Entomology* **36**: 229–240.

**Gullan PJ, Vranjic JA**. **1991**. The taxonomy of the gum tree scales *Eriococcus confusus* Maskell and *E. coriaceus* Maskell (Hemiptera: Coccoidea: Eriococcidae). *General and Applied Entomology* **23**: 21–40.

**Hardy NB, Beardsley Jr. JW, Gullan PJ**. **2011**. Uncovering diversity of Australian *Eucalyptus*-constrained felt scales (Hemiptera: Coccoidea: Eriococcidae). *Systematic Entomology* **36**: 497–528.

**Hardy NB, Beardsley Jr JW, Gullan PJ**. **2019**. A revision of *Lachnodius* Maskell (Hemiptera, Coccomorpha, Eriococcidae). *ZooKeys* **818**: 43–88.

**Hardy NB, Gullan PJ**. **2007**. A new genus and four new species of felt scales on *Eucalyptus* (Hemiptera: Coccoidea: Eriococcidae) in south-eastern Australia. *Australian Journal of Entomology* **46**: 106–120.

**Hardy NB, Gullan PJ**. **2008**. *Opisthoscelis nigra* Froggatt (Eriococcidae) is an armored scale *Maskellia nigra* (Froggatt) (Diaspididae) (Hemiptera: Coccoidea). In: *Proceedings of the XI International Symposium on Scale Insect Studies*. Oeiras, Portugal: ISA Press Lisbon, 59–62.

**Hardy NB, Gullan PJ**. **2010**. Australian gall-inducing scale insects on *Eucalyptus*: revision of *Opisthoscelis* Schrader (Coccoidea, Eriococcidae) and descriptions of a new genus and nine new species. *ZooKeys* **58**: 1–74.

**Henderson RC**. **2011**. Diaspididae (Insecta: Hemiptera: Coccoidea). *Fauna of New Zealand* **66**: 1–275.

**Hoare RJB, Johansson R, van Nieukerken EJ, Nielsen ES**. **1997**. Australian Nepticulidae (Lepidoptera): redescription of the named species. *Insect Systematics & Evolution* **28**: 1–26.

**Hoare RJB, van Nieukerken EJ**. **2013**. Phylogeny and host-plant relationships of the Australian Myrtaceae leafmining moth genus *Pectinivalva* (Lepidoptera, Nepticulidae), with new subgenera and species. *ZooKeys* **278**: 1–64.

**Hodgson CJ, Williams DJ**. **2016**. A revision of the family Cerococcidae Balachowsky (Hemiptera: Sternorrhyncha, Coccomorpha) with particular reference to species from the Afrotropical, western Palaearctic and western Oriental Regions, with the revival of *Antecerococcus* Green and description of a new genus and fifteen new species, and with ten new synonomies. *Zootaxa* **4091**: 1–175.

**Howell JO, Kosztarab MP**. **1972**. *Morphology and systematics of the adult females of the genus* Lecanodiaspis *(Homoptera: Coccoidea: Lecanodiaspididae)*. Blacksburg, U.S.A.: Research Division Bulletin Virginia Polytechnic Institute and State University.

**Hoy JM**. **1962**. Eriococcidae (Homoptera: Coccoidea) of New Zealand. *New Zealand Department of Scientific and Industrial Research Bulletin* **146**: 1–219.

**Hoy JM**. **1963**. A catalogue of the Eriococcidae (Homoptera: Coccoidea) of the world. *New Zealand Department of Scientific and Industrial Research Bulletin* **150**: 1–260.

**Hudson NM**. **1967**. A list of the armoured scale insects (Diaspididae) and their hosts in Tasmania. *Papers and Proceedings of the Royal Society of Tasmania* **101**: 91–95.

**Ikeda E**. **1999**. A revision of the world species of *Quadrastichodella* Girault, with descriptions of four new species (Hymenoptera, Eulophidae). *Insecta Matsumarana* **55**: 13–35.

**Jones DL, Elliot WR, Jones SR**. **2015**. *Pests, Diseases, Ailments and Allies of Australian Plants*. Chatswood, Australia: Reed New Holland.

**Kile GA, Hardy RJ, Turnbull CRA**. **1979**. The Association Between *Abantiades latipennis* (Lepidoptera, Family Hepialidae) and *Eucalyptus obliqua* and *Eucalyptus regnans* in Tasmania. *Australian Journal of Entomology* **18**: 7–17.

**Kim I-K, La Salle J**. **2008**. A new genus and species of Tetrastichinae (Hymenoptera: Eulophidae) inducing galls in seed capsules of *Eucalyptus*. *Zootaxa* **1745**: 63–68.

**Kim I-K, McDonald M, La Salle J**. **2005**. *Moona*, a new genus of tetrastichine gall inducers (Hymenoptera: Eulophidae) on seeds of *Corymbia* (Myrtaceae) in Australia. *Zootaxa* **989**: 1–10.

**La Salle J, Arakelian G, Garrison RW, Gates MW**. **2009**. A new species of invasive gall wasp (Hymenoptera: Eulophidae: Tetrastichinae) on blue gum (*Eucalyptus globulus*) in California. *Zootaxa* **2121**: 35–43.

**Laing F**. **1925**. Descriptions of some new genera and species of Coccidae. *Bulletin of Entomological Research* **16**: 51–66.

**Laing F**. **1929**. Report on Australian Coccidae. *Bulletin of Entomological Research* **20**: 15–37.

**Le NH, Nahrung HF, Griffiths M, Lawson SA**. **2018**. Invasive *Leptocybe* spp. and their natural enemies: Global movement of an insect fauna on eucalypts. *Biological Control* **125**: 7–14.

**Le NH, Nahrung HF, Morgan JAT, Lawson SA**. **2020**. Multivariate ratio analysis and DNA markers reveal a new Australian species and three synonymies in eucalypt-gall-associated *Megastigmus* (Hymenoptera: Megastigmidae). *Bulletin of Entomological Research* **110**: 709–724.

**LeBreton M, Vaarwerk M**. **1993**. Miscellaneous notes on *Apiomorpha* spp. (Homoptera: Eriococcidae) and their host plants in New South Wales. *Sydney Basin Naturalist* **2**: 25–29.

**LeBreton M, Vaarwerk M**. **1994**. Records of three *Apiomorpha* Rübsaaman (Homoptera: Eriococcidae: Apiomorphinae) employing *Eucalyptus burgessiana* as a host in the Blue Mountains, N.S.W. *Sydney Basin Naturalist* **1**: 77–78.

**Leonardi G**. **1900**. Generi e specie di diaspiti. Saggio di sistematica degli *Aspidiotus*. *Rivista di Patologia Vegetale* **8**: 298–363.

**Lindinger L**. **1913**. Coccidae. In: Michaelsen W, Hartmeyer R, eds. *Die Fauna südwest-Australiens. Ergebnisse der Hamburger südwest-australischen Forschungsreise* 1905. Jena, Germany: Gustav Fischer Verlag, 343–348.

**Marlatt CL**. **1908**. The genus *Pseudaonidia*. *Proceedings of the Entomological Society of Washington* **9**: 131–141.

**Maskell WM**. **1879**. On some Coccidae in New Zealand. *Transactions and Proceedings of the New Zealand Institute* **11**: 187–228.

**Maskell WM**. **1889**. On some new South Australian Coccidae. *Transactions of the Royal Society of South Australia* **11**: 101–111.

**Maskell WM**. **1890a**. On some species of Psyllidae in New Zealand. *Transactions and Proceedings of the New Zealand Institute* **22**: 157–168.

**Maskell WM**. **1890b**. Further notes on Coccidae, with descriptions of new species from Australia, Fiji, and New Zealand. *Transactions and Proceedings of the New Zealand Institute* **22**: 133–156.

**Maskell WM**. **1892**. Further coccid notes: with descriptions of new species, and remarks on coccids from New Zealand, Australia and elsewhere. *Transactions and Proceedings of the New Zealand Institute* **24**: 1–64.

**Maskell WM**. **1893**. Further coccid notes: with descriptions of new species from Australia, India, Sandwich Islands, Demerara, and South Pacific. *Transactions and Proceedings of the New Zealand Institute* **25**: 201–252.

**Maskell WM**. **1894**. Further coccid notes with descriptions of several new species and discussion of various points of interest. *Transactions and Proceedings of the New Zealand Institute* **26**: 65–105.

**Maskell WM**. **1895**. Further coccid notes: with description of new species from New Zealand, Australia, Sandwich Islands, and elsewhere, and remarks upon many species already reported. *Transactions and Proceedings of the New Zealand Institute* **27**: 36–75.

**Maskell WM**. **1896**. Further coccid notes, with descriptions of new species and discussions of questions of interest. *Transactions and Proceedings of the New Zealand Institute* **28**: 380–411.

**Maskell WM**. **1897**. Further coccid notes: with descriptions of new species and discussions of points of interest. *Transactions and Proceedings of the New Zealand Institute* **29**: 293–331.

**Maskell WM**. **1898**. Further coccid notes: with descriptions of new species, and discussion of points of interest. *Transactions and Proceedings of the New Zealand Institute* **30**: 219–252.

**Mayo GM, Austin AD, Adams M**. **1997**. Morphological and electrophoretic taxonomy of the Australian eucalypt leaf-blister sawfly genus *Phylacteophaga* (Hymenoptera: Pergidae): a potential major pest group of eucalypts worldwide. *Bulletin of Entomological Research* **87**: 595–608.

**Mazanec Z**. **1967**. Mortality and Diameter Growth in Mountain Ash Defoliated by Phasmatids. *Australian Forestry* **31**: 221–223.

**Mazanec Z**. **1974**. Influence of Jarrah Leaf Miner on the Growth of Jarrah. *Australian Forestry* **37**: 32–42.

**Mazanec Z**. **1978**. A sampling scheme for estimating population density of the jarrah leaf miner, *Perthida glyphopa* (Lepidoptera: Incurvariidae). *Australian Journal of Entomology* **17**: 275–285.

**Mazanec Z**. **1981**. The jarrah leaf miner and its natural enemies. *Journal of the Department of Agriculture, Western Australia, Series 4*: 4.

**Mazanec Z**. **1983**. The immature stages and life history of the jarrah leafminer, *Perthida glyphopa* Common (Lepidoptera: Incurvariidae). *Australian Journal of Entomology* **22**: 101–108.

**Mazanec Z**. **1985**. Resistance of *Eucalyptus marginata* to *Perthida glyphopa* (Lepidoptera: Incurvariidae). *Australian Journal of Entomology* **24**: 209–221.

**Mazanec Z**. **1987**. Natural enemies of *Perthida glyphopa* Common (Lepidoptera: Incurvariidae). *Australian Journal of Entomology* **26**: 303–308.

**Mazanec Z**. **1988**. Immature stages and life history of *Chrysonotomyia* sp. c (Hymenoptera: Eulophidae), a parasitoid of the jarrah leafminer. *Australian Journal of Entomology* **27**: 279–292.

**Mazanec Z**. **1990a**. The immature stages and life history of *Diaulomorpha* sp. (Hymenoptera: Eulophidae), a parasitoid of *Perthida glyphopa* Common (Lepidoptera: Incurvariidae). *Australian Journal of Entomology* **29**: 147–159.

**Mazanec Z**. **1990b**. The immature stages and biology of *Chrysonotomyia* sp. a (Hymenoptera: Eulophidae), a parasitoid of *Perthida glyphopa* Common (Lepidoptera: Incurvariidae). *Australian Journal of Entomology* **29**: 139–146.

**Mazanec Z, Justin MJ**. **1986**. Oviposition behaviour and dispersal of *Perthida glyphopa* Common (Lepidoptera: Incurvariidae). *Australian Journal of Entomology* **25**: 149–160.

**Mazanec Z, Justin MJ**. **1994**. Fecundity and Oviposition by *Perthida glyphopa* Common (Lepidoptera: Incurvariidae). *Australian Journal of Entomology* **33**: 223–234.

**McKenzie HL**. **1950**. The genera *Lindingaspis* Macgillivray and *Marginaspis* Hall (Homoptera; Coccoidea; Diaspididae). (Contribution No. 69). *Microentomology* **15**: 98–124.

**Mendel Z, Protasov A, Fisher N, La Salle J**. **2004**. Taxonomy and biology of *Leptocybe invasa* gen. & sp. n. (Hymenoptera: Eulophidae), an invasive gall inducer on *Eucalyptus*. *Australian Journal of Entomology* **43**: 101–113.

**Miller DR**. **1970**. A new genus and species of scale insect from Tasmania (Homoptera: Eriococcidae). *Journal of the Australian Entomological Society* **9**: 157–159.

**Mills PJ, Cook LG**. **2010**. Disparity in chromosomal variation within the *Apiomorpha minor* species-group. *Entomologia Hellenica* **19**: 82–89.

**Mills PJ, Cook LG**. **2014**. Rapid chromosomal evolution in a morphologically cryptic radiation. *Molecular Phylogenetics and Evolution* **77**: 126–135.

**Mills PJ, Gullan PJ, Cook LG**. **2017**. Nomenclatural changes in the Australasian gall-inducing genus *Apiomorpha* Rübsaamen (Hemiptera: Coccomorpha: Eriococcidae). *Zootaxa* **4250**: 484–488.

**Mills PJ, MacDonald ML, Rigby LM, Cook LG**. **2011**. A recently discovered species of *Apiomorpha* Rübsaamen (Hemiptera: Coccoidea: Eriococcidae) with unusual gall morphology. *Zootaxa* **3093**: 55–63.

**Mills PJ, Semple TL, Garland KLS, Cook LG**. **2016**. Two recently discovered species of *Apiomorpha* (Hemiptera: Eriococcidae) feeding on eudesmid eucalypts in Western Australia reaffirm host conservatism in this gall-inducing scale insect genus. *Invertebrate Systematics* **30**: 255–273.

**Molina-Mercader G, Angulo AO, Olivares TS, Sanfuentes E, Castillo-Salazar M, Rojas E, Toro-Núñez O, Benítez HA, Hasbún R**. **2019**. *Ophelimus migdanorum* Molina-Mercader sp. nov. (Hymenoptera: Eulophidae): Application of integrative taxonomy for disentangling a polyphenism case in *Eucalyptus globulus* Labill forest in Chile. *Forests* **10**: 720.

**Moore KM**. **1959**. Observations on some Australian forest insects. 4. *Xyleborus truncatus* Erichson 1842 (Coleoptera: Scolytidae) associated with dying *Eucalyptus saligna* Smith (Sydney blue-gum). *Proceedings of the Linnean Society of New South Wales* **84**: 186–193.

**Moore KM**. **1961a**. Observations on some Australian forest insects. 7. The significance of the *Glycaspis* spp. (Hemiptera: Homoptera, Psyllidae) associations with their *Eucalyptus* spp. hosts; erection of a new subgenus and descriptions of thirty-eight new species of *Glycaspis*. *Proceedings of the Linnean Society of New South Wales* **86**: 128–167.

**Moore KM**. **1961b**. Observations on some Australian forest insects. 8. The biology and occurrence of *Glycaspis baileyi* Moore in New South Wales. *Proceedings of the Linnean Society of New South Wales.* **86**: 185–200.

**Moore KM**. **1961c**. Observations on some Australian forest insects. 9. A new species of *Glycaspis* (*Glycaspis*) (Homoptera: Psyllidae). *Proceedings of the Linnean Society of New South Wales* **86**: 128–167.

**Moore KM**. **1963**. Observations on some Australian forest insects. 11. Two species of lepidopterous leaf-miners attacking *Eucalyptus pilularis* Smith. *Australian Zoologist* **13**: 46–53.

**Moore KM**. **1966**. Observations on some Australian forest insects. 22. Notes on some Australian leaf-miners. *Australian Zoologist* **13**: 303–349.

**Moore KM**. **1970**. Observations on some Australian forest insects. 23. A revision of the genus *Glycaspis* (Homoptera: Psyllidae) with descriptions of seventy-three new species. *Proceedings of the Linnean Society of New South Wales* **15**: 248–342.

**Moore KM**. **1972**. Observations on some Australian forest insects. 26. Some insects attacking three important tree species. *Australian Zoologist* **17**: 30–39.

**Moore KM**. **1984**. Two new species of *Glycaspis* (Homoptera: Psylloidea) from tropical Queensland, with notes on the genus. *Proceedings of the Linnean Society of New South Wales* **107**: 475–478.

**Morgan FD, Bungey RS**. **1981**. Dynamics of population outbreaks of Psyllidae (Lerp Insects) on eucalypts. In: *Eucalypt Dieback in Forests and Woodlands*. Melbourne, Australia: CSIRO Publishing, 127–129.

**Morrison H, Morrison ER**. **1923**. The scale insects of the subfamilies Monophlebinae and Margarodinae treated by Maskell. *Proceedings of the United States National Museum* **62**: 1–47.

**Morrison H, Morrison ER**. **1927**. The Maskell species of scale insects of the subfamily Asterolecaniinae. *Proceedings of the United States National Museum* **71**: 1–67.

**Neumann FG, Harris JA, Wood CH**. **1977**. *The Phasmatid Problem in Mountain Ash Forests of the Central Highlands of Victoria*. Melbourne, Australia: Forestry Comission, Victoria.

**Normark BB, Okusu A, Morse GE, Peterson DA, Itioka T, Schneider SA**. **2019**. Phylogeny and classification of armored scale insects (Hemiptera: Coccomorpha: Diaspididae). *Zootaxa* **4616**: 1–98.

**Paine TD, Steinbauer MJ, Lawson SA**. **2011**. Native and exotic pests of *Eucalyptus*: a worldwide perspective. *Annual Review of Entomology* **56**: 181–201.

**Palzer C**. **1981**. Aetiology of gully dieback. In: Old KM, Kile GA, Ohmart CP, eds. *Eucalypt Dieback in Forests and Woodlands*. Melbourne, Australia: CSIRO Publishing, 174–178.

**Phillips CL**. **1993**. Insect pest problems of eucalypt plantations in Australia. 5. South Australia. *Australian Forestry* **56**: 378–380.

**Pook EW**. **1981**. Drought and dieback of eucalypts in dry sclerophyll forests and woodlands of the Southern Tablelands, New South Wales. In: Old KM, Kile GA, Ohmart CP, eds. *Eucalypt Dieback in Forests and Woodlands*. Melbourne, Australia: CSIRO Publishing, 179–189.

**Protasov A, La Salle J, Blumberg D, Brand D, Saphir N, Assael F, Fisher N, Mendel Z**. **2007**. Biology, revised taxonomy and impact on host plants of *Ophelimus maskelli*, an invasive gall inducer on *Eucalyptus* spp. in the Mediterranean Area. *Phytoparasitica* **35**: 50–76.

**Qin TK, Gullan PJ**. **1989**. *Cryptostigma* Ferris: a coccoid genus with a strikingly disjunct distribution (Homoptera: Coccidae). *Systematic Entomology* **14**: 221–232.

**Qin TK, Gullan PJ**. **1992**. A revision of the Australian pulvinariine soft scales (Insecta: Hemiptera: Coccidae). *Journal of Natural History* **26**: 103–164.

**Qin TK, Gullan PJ**. **1994**. Taxonomy of the wax scales (Hemiptera: Coccidae: Ceroplastinae) in Australia. *Invertebrate Taxonomy* **8**: 923–959.

**Raman A, Withers TM**. **2003**. Oviposition by introduced *Ophelimus eucalypti* (Hymenoptera: Eulophidae) and morphogenesis of female-induced galls on *Eucalyptus saligna* (Myrtaceae) in New Zealand. *Bulletin of Entomological Research* **93**: 55–63.

**Reid C a. M**. **2006**. A taxonomic revision of the Australian Chrysomelinae, with a key to the genera (Coleoptera: Chrysomelidae). *Zootaxa* **1292**: 1–119.

**Sanders JG**. **1906**. Catalogue of recently described Coccidae. *Technical Series of the Bureau of Entomology of the United States Department of Agriculture* **12**: 1–18.

**Schrader HL**. **1863**. Further communication on the gall-making Coccidae. *Transactions of the Entomological Society of New South Wales* **1**: 6–8.

**Spencer KA**. **1963**. The Australian Agromyzidae (Diptera, Insecta). *Records of the Australian Museum* **25**: 305–354.

**Spencer KA**. **1977**. *A revision of the Australian Agromyzidae (Diptera)*. Perth, Australia: Western Australian Museum.

**Szent-Ivany SH, Womersley JS**. **1962**. First record of the gall-forming coccid genus *Apiomorpha*-Ruebsaamen (Hemiptera, Coccidae) in Papua. *Transactions of the Papua and New Guinea Science Society* **3**: 20–22.

**Takagi S**. **1984**. Some aspidiotine scale insects with enlarged setae on the pygidial lobes (Homoptera: Coccoidea: Diaspididae). *Insecta Matsumurana* **28**: 1–69.

**Tanton M, Epila J**. **1984**. Parasitization of Larvae of *Paropsis atomaria* Ol. (Coleoptera: Chrysomelidae) in the Australian Capital Territory. *Australian Journal of Zoology* **32**: 251.

**Taylor KL**. **1962**. The Australian genera *Cardiaspina* Crawford and *Hyalinaspis* Taylor, (Homoptera: Psyllidae). *Australian Journal of Zoology* **10**: 307.

**Taylor KL**. **1984**. A new genus and three new species of Spondyliaspidae (Homoptera: Psylloidea) on *Eucalyptus*. *Australian Journal of Entomology* **23**: 13–19.

**Taylor KL**. **1985**. Australian psyllids: a new genus of Ctenarytainini (Homoptera: Psylloidea) on *Eucalyptus*, with nine new species. *Australian Journal of Entomology* **24**: 17–30.

**Taylor KL**. **1987**. Revision of *Eucalyptolyma* Froggatt (Homoptera: Psylloidea) with two new genera of Australian psyllids. *Australian Journal of Entomology* **26**: 97–127.

**Taylor KL**. **1997**. A new Australian species of *Ctenarytaina* Ferris and Klyver (Hemiptera: Psyllidae: Spondyliaspidinae) established in three other countries. *Australian Journal of Entomology* **36**: 113–115.

**Tepper JGO**. **1893**. Descriptions of South Australian brachyscelid galls. *Transactions of the Royal Society of South Australia* **17**: 265–280.

**Timberlake PH**. **1957**. A new entedontine Chalcid-fly from seed capsules of *Eucalyptus* in California (Hymenoptera: Eulophidae). *The Pan-Pacific Entomologist* **33**: 109–110.

**Tribe GD, Cillie JJ**. **1997**. Biology of the Australian tortoise beetle *Trachymela tincticollis* (Blackburn) (Chrysomelidae: Chrysomelini: Paropsina), a defoliator of *Eucalyptus* (Myrtaceae), in South Africa. *African Entomology* **5**: 109–123.

**Wallace MMH**. **1970**. The biology of the jarrah leaf miner, *Perthida glyphopa* Common (Lepidoptera: In­curvariidae). *Australian Journal of Zoology* **18**: 91–104.

**Whitham TG, Morrow PA, Potts BM**. **1994**. Plant hybrid zones as centers of biodiversity: the herbivore community of two endemic Tasmanian eucalypts. *Oecologia* **97**: 481–490.

**Williams DJ**. **1985**. *Australian mealybugs*. London, UK: British Museum (Natural History).

**Williams DJ**. **1987**. *Rhizoecus* (Insecta: Homoptera: Pseudococcidae) from Australia with a description of a new species damaging garden plants in Victoria. *Memoirs of the Museum of Victoria* **48**: 191–194.

**Williams DJ**. **1989**. The mealybug genus *Rastrococcus* Ferris (Hemiptera: Pseudococcidae). *Systematic Entomology* **14**: 433–486.

**Williams ML, Kosztarab MP**. **1970**. *A morphological and systematic study of the first instar nymphs of the genus Lecanodiaspis (Homoptera: Coccoidea: Lecanodiaspididae)*. Blacksburg, U.S.A.: Research Division Bulletin Virginia Polytechnic Institute and State University.

**Wylie FR, Bevege DI**. **1981**. Eucalypt Diebacks in Queensland. In: Old KM, Kile GA, Ohmart CP, eds. *Eucalypt Dieback in Forests and Woodlands*. Melbourne, Australia: CSIRO Publishing, 31–36.

**Yang M-M, Lin Y-C, Wu Y, Fisher N, Saimanee T, Sangtongpraow B, Zhu C, Chiu WC-H, Salle JL**. **2014**. Two new *Aprostocetus* species (Hymenoptera: Eulophidae: Tetrastichinae), fortuitous parasitoids of invasive eulophid gall inducers (Tetrastichinae) on *Eucalyptus* and *Erythrina*. *Zootaxa* **3846**: 261.

**Zondag R**. **1977**. *Eriococcus coriaceus* Maskell (Hemiptera: Coccoidea: Eriococcidae), In: *Forest and Timber Insects in New Zealand, No. 22*. Rotorua, New Zealand: Forest Research Institute and New Zealand Forest Service.
